# Eco-Friendly and Reusable CNT/Halloysite-Based Sensor for Electrochemical Ciprofloxacin Detection

**DOI:** 10.3390/s26217086

**Published:** 2026-11-09

**Authors:** Carlo Spampinato, Ivana Maria Caruso, Viviana Scuderi, Simona Filice, Andrzej Krasiński, Silvia Scalese

**Affiliations:** 1 Consiglio Nazionale delle Ricerche—Istituto di Microelettronica e Microsistemi (CNR-IMM), VIII Strada n. 5, Zona Industriale, 95121 Catania, Italy; ivana.caruso@phd.unict.it (I.M.C.); simona.filice@cnr.it (S.F.); silvia.scalese@cnr.it (S.S.); 2 Dipartimento di Fisica ed Astronomia, Università degli Studi di Catania, Via Santa Sofia n. 64, 95125 Catania, Italy; 3 Faculty of Chemical and Process Engineering, Warsaw University of Technology, Warynskiego 1, 00-645 Warsaw, Poland; andrzej.krasinski@pw.edu.pl

**Keywords:** ciprofloxacin, carbon nanotubes, halloysite nanotubes, electrochemical sensor, screen-printed electrode, sustainable sensing, reusable sensor

## Abstract

Antibiotic residues in aquatic environments require sensing approaches that are not only analytically effective but also compatible with aqueous fabrication and reusable after regeneration. Here, we report a ciprofloxacin (CIP) electrochemical sensor based on commercial carbon screen-printed electrodes modified with carbon nanotubes (CNTs) and halloysite nanotubes (HTs). The modifier is prepared and deposited entirely from aqueous dispersions: CNTs provide conductive pathways, whereas naturally occurring halloysite supplies an inexpensive adsorptive phase that may favor local analyte adsorption. The optimized sensing layer exhibited a mean sensitivity of $0.2916\;\mu A\;\mu M^{-1}$ over the 4.2–34μM calibration range, with a relative standard deviation of 4.29% across three independently fabricated electrodes. An estimated limit of detection (LOD) of approximately 34 nM was calculated from the blank signal variability and calibration sensitivity. Measurements were intentionally performed at pH 7.0, representative of near-neutral aquatic matrices and avoiding acidification pretreatment. Although mildly acidic conditions may further favor CIP adsorption, the neutral-pH workflow provides a conservative and more sustainability-oriented assessment. Following sensing, a 10min UVA-assisted cleaning treatment reduced the residual CIP-related signal and enabled subsequent electrode reuse. The regenerated electrode retained approximately 82.4% of its initial sensitivity in the investigated lower concentration interval (4.2–17 μM), demonstrating substantial recovery of its analytical response after UVA treatment.

## 1. Introduction

The widespread use and discharge of pharmaceutical compounds into the environment raise significant concerns because of their persistence, ecological effects, and contribution to antimicrobial resistance [1]. Among these contaminants, ciprofloxacin (CIP), a second-generation fluoroquinolone antibiotic widely used to treat bacterial infections, is frequently detected in wastewater, surface water, and groundwater at trace levels [2,3]. Even residual amounts of CIP, often reported in concentrations from ng ${\mathrm{mL}}^{-1}$ to μg ${\mathrm{mL}}^{-1}$ [4], can contribute to the selection and spread of resistant bacterial strains and disturb aquatic ecosystems, making accessible monitoring tools important for environmental protection [5,6,7].

Conventional analytical techniques, including high-performance liquid chromatography (HPLC), chromatography coupled with mass spectrometry (GC/LC-MS), nuclear magnetic resonance (NMR), and spectroscopic methods, provide accurate antibiotic quantification but generally require dedicated instrumentation, trained personnel, and sample-preparation steps, e.g., solid-phase extraction that limits decentralized monitoring [8]. Electrochemical sensors are attractive alternatives because they can combine rapid response, low cost, compact instrumentation, and compatibility with screen-printed formats [5,9,10].

Several electrochemical sensing strategies have been developed for CIP detection, exploiting different electrode materials and recognition mechanisms. Carbon-based platforms, including CNT-based composites and graphene-modified electrodes, have demonstrated promising analytical performance and low detection limits [11,12]. Alternatively, aptamer-functionalized electrodes and molecularly imprinted polymers (MIPs) have been investigated to improve molecular recognition and selectivity [13,14]. Metal-containing nanomaterials, including copper–zinc ferrites and metal–organic frameworks (MOFs)/metal oxide composites, have also been employed to enhance the electrochemical response toward CIP [15,16]. Although several of these approaches achieve very low detection limits, they may involve additional functionalization steps, complex material preparation, or specific recognition elements. These developments highlight the importance of balancing analytical performance with fabrication simplicity and sensor reusability.

However, analytical sensitivity is only one aspect of sensor sustainability. Many nanostructured electrochemical platforms rely on organic solvents, hazardous reagents, multistep fabrication, or difficult-to-recover nanomaterials [6]. Moreover, screen-printed sensors are commonly treated as disposable devices: strong adsorption of antibiotics and other organic contaminants can irreversibly foul the sensing interface after a measurement, increasing material consumption and the waste generated per analysis. A useful environmental sensor should therefore aim not only for adequate analytical performance, but also for low-impact fabrication and, where possible, regeneration and reuse.

Here, commercial carbon screen-printed electrodes (SPE_C) are modified with carbon nanotubes (CNTs) and halloysite nanotubes (HTs) using water as the sole processing solvent. CNTs provide high electrical conductivity, a large accessible surface, and efficient pathways for interfacial electron transfer [17,18]. Halloysite is a naturally occurring tubular clay mineral of the kaolin group, formed by rolled 1:1 aluminosilicate layers and characterized by chemically distinct inner and outer surfaces and a high density of adsorption sites [19,20,21]. Its outer Si–O/Si–OH-rich surface is typically negatively charged under near-neutral conditions, whereas the Al–OH-rich lumen can retain a different charge character. This amphoteric chemistry is particularly relevant for CIP because its speciation changes with pH: the molecule is mainly cationic below $pK_{a1}\approx 6.1$, predominantly zwitterionic around neutral pH, and increasingly anionic above $pK_{a2}\approx 8.6$–8.7 [22]. The working hypothesis is therefore that the CNT network maintains efficient electronic pathways, while halloysite may facilitate local CIP adsorption through different surface interactions, including electrostatic attraction and possible complexation involving Al–OH sites. The relative contribution of these mechanisms remains to be established [23,24].

**Figure 1:**
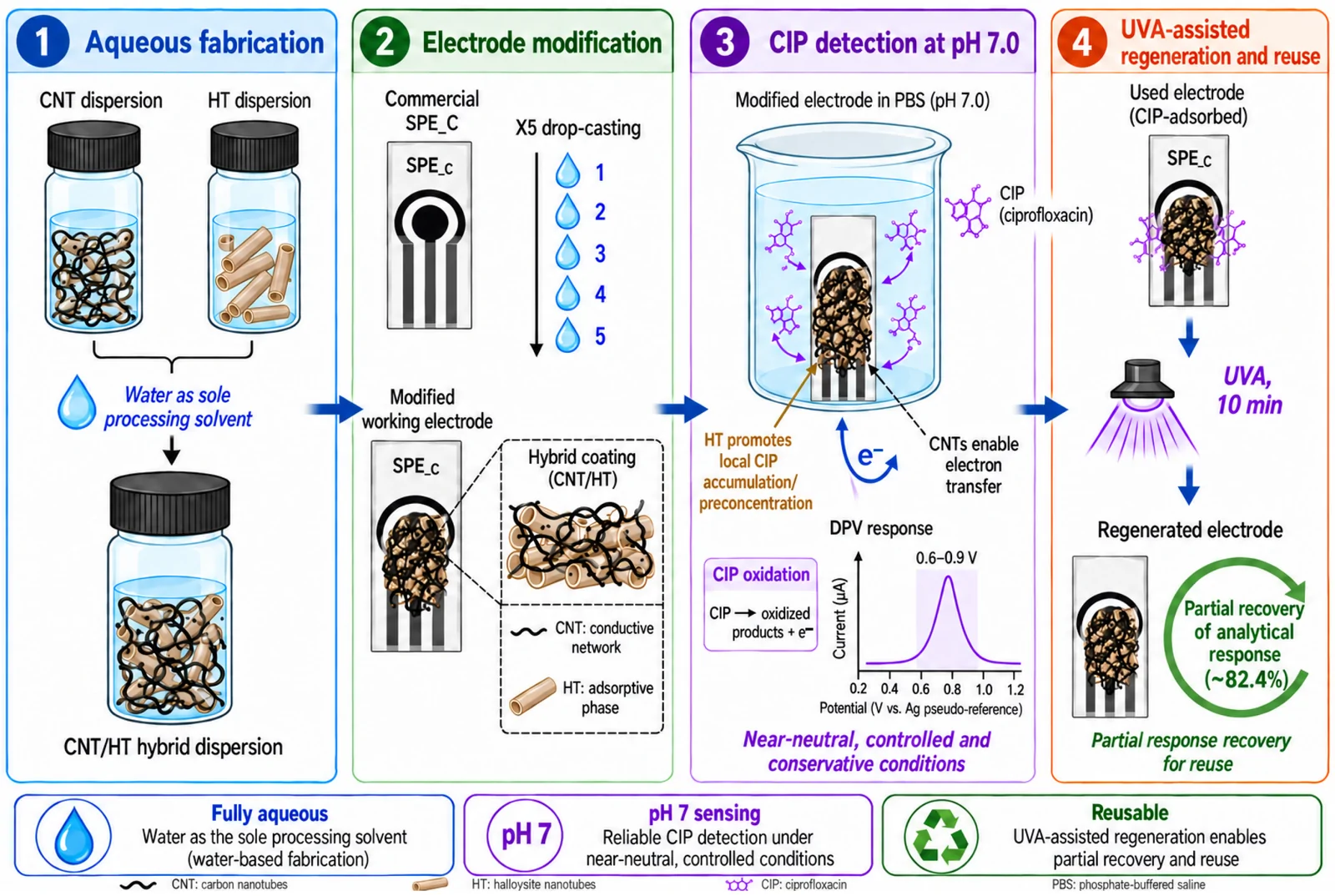
Schematic representation of the proposed CNT/halloysite-based electrochemical sensing platform. The sensor is fabricated entirely from aqueous CNT and HT dispersions by sequential drop-casting onto a commercial screen-printed carbon electrode. During CIP detection at pH 7.0, halloysite provides an adsorptive phase that promotes local analyte adsorption, while the CNT network facilitates electron transfer and the electrochemical oxidation of CIP. After measurement, UVA irradiation for 10min promotes removal or transformation of adsorbed CIP species, enabling partial recovery of the analytical response and subsequent electrode reuse.

The main contribution of this work is consequently not a record-low detection limit, but the integration of three features in a simple architecture: fully aqueous electrode modification, an analytically estimated nanomolar detection limit for CIP using commercially available carbon/clay materials, and UVA-assisted cleaning that enables partial recovery of the sensing response after adsorption-induced fouling. The resulting electrodes were investigated by scanning electron microscopy (SEM), energy-dispersive X-ray spectroscopy (EDX), electrochemical impedance spectroscopy (EIS), and differential pulse voltammetry (DPV). Particular attention is given to the balance between HT loading and CNT-mediated charge transport and to the recovery of the analytical response after UVA-assisted cleaning. This combination directly addresses both the solvent footprint of nanomaterial processing and the waste burden associated with single-use antibiotic sensors. The overall sensing strategy and the complementary roles of the CNT and HT components are summarized in Figure 1, highlighting the fully aqueous fabrication route, CIP detection under near-neutral conditions, and UVA-assisted regeneration of the sensing interface for repeated use.

## 2. Materials and Methods

### 2.1. Chemicals and Materials

Multi-walled carbon nanotubes (CNTs) with average diameter/length of 9.5 nm/1.5 μm, respectively, and carboxylic acid functionalization, were purchased at Sigma-Aldrich S.r.l. (Milan, Italy). The –COOH functional groups present on carbon nanotubes allow to get a better dispersion in solution. Halloysite nanotubes were obtained from Sigma-Aldrich (CAS No. 1332-58-7, product no. 685445, Saint Louis, USA) and employed without further purification. Ciprofloxacin (CIP) and tinidazole (TNZ) were purchased from Merck (Darmstadt, Germany). Disposable carbon screen-printed membrane electrodes (DRP-110-U75, Metrohm DropSens, Oviedo, Spain) were used as the electrochemical transducers throughout this work. These electrodes incorporate a carbon working electrode, a carbon counter electrode, and an integrated silver (Ag) pseudo-reference electrode. All potentials reported in this study are expressed versus this Ag pseudo-reference, without conversion to the Ag/AgCl scale.

### 2.2. Preparation of CNT and HT Nanocomposite

Aqueous dispersions were prepared using ultrapure water as the solvent. In all cases, the suspensions were sonicated in an ultrasonic bath for 1 h to promote deagglomeration and improve dispersion stability.

A stock CNT suspension was obtained by dispersing 0.06 mg of CNTs in 1 mL of water, using an ultrasonic agitator for 1 hour (CNT stock). Similarly, a stock halloysite dispersion was prepared by dispersing 0.5 mg of halloysite nanotubes in 1 mL of water (HT stock). Two halloysite working dispersions were then prepared by diluting the HT stock with water to obtain: (i) solution A, with a volumetric ratio of 1 part HT stock to 9 parts water (1:10, *v*/*v*), and (ii) solution B, with a volumetric ratio of 1 part HT stock to 49 parts water (1:50, *v*/*v*).

Hybrid modifier inks were prepared by mixing the CNT stock dispersion with the halloysite working dispersions at a 1:1 volumetric ratio. The resulting mixtures were denoted as CNT-HTA (CNT stock + dispersion A) and CNT-HTB (CNT stock + dispersion B), respectively.

For reference experiments, two additional dispersions were prepared and used as controls: (i) CNT reference, obtained by diluting the CNT stock with water at a 1:1 ratio, achieving a CNT concentration of 0.03 mg/mL (suspension without HT); (ii) HTA, obtained by diluting solution A with water at a 1:1 ratio (HT 0.025 mg/mL), to obtain an HT-only reference dispersion (without CNT) at the same HT concentration (0.025 mg/mL) as the CNT–HTA mixture.All dispersions were freshly prepared before electrode modification.

### 2.3. Modification of the SPE_C Electrodes

Commercial carbon screen-printed electrodes (SPE_C, DRP-110-U75) were modified by drop-casting the prepared dispersions onto the working electrode surface, as listed in Table 1. For each formulation (CNT, HTA, CNT-HTA, and CNT-HTB), an aliquot of 10 μL was pipetted directly onto the carbon working electrode and dried under vacuum at $25^{\circ }$C for 15 min to obtain the modifier layer.

To investigate the effect of the deposited loading, additional electrodes were prepared using multiple sequential drop-casting cycles for the CNT, CNT-HTA, and CNT-HTB based modifiers. In these cases, 10 µL of the corresponding dispersion was deposited repeatedly for either 3 or 5 cycles. After each deposition, the electrode was dried under vacuum under the same conditions ($25^{\circ }$C for 15 min) before applying the subsequent drop, thereby promoting reproducible layer build-up and increasing the overall amount of material immobilized on the electrode surface. Table 1 reports the list and specifications of the prepared modified electrodes.

### 2.4. Sample Characterization and Regeneration

The SEM analyses were performed with a Zeiss FE-SEM Supra 3 Microscope operating at 2–3 kV equipped with an energy-dispersive X-ray (EDX) microanalysis system (X-Max, 80 ${\mathrm{mm}}^{2}$ by Oxford Instruments, Abingdon, UK). EIS measurements were performed in a 10 mM $K_{3}$[Fe(CN)_6_] electrolyte. EIS spectra were recorded over the 100 kHz-0.1 Hz frequency range (61 points) by applying a small-signal sinusoidal perturbation ($E_{\mathrm{ac}}=10$ mV, rms) at a fixed DC bias ($E_{\mathrm{dc}}$) selected close to the formal potential/peak potential of the redox probe, in order to probe the interfacial charge-transfer process under quasi-equilibrium conditions. The EIS spectra were fitted using the equivalent-circuit fitting functionality implemented in NOVA 2.1 software (Metrohm Autolab Heriseau, Switzerland). A Randles-type equivalent circuit, comprising the solution resistance, charge-transfer resistance (Rct), a constant phase element, and a Warburg diffusion contribution, was employed, as illustrated in Figure S2. The same equivalent-circuit model was applied to all investigated electrodes to ensure a consistent comparison of the fitted electrochemical parameters. The experimental impedance data and corresponding fitted curves are reported in Figure S2, while the extracted parameters are summarized in Table 2. For spectra displaying incomplete semicircular features, Rct was considered an apparent, model-dependent parameter rather than a directly resolved charge-transfer resistance. DPV measurements for ciprofloxacin were carried out in 0.10 M phosphate-buffered saline (PBS) at pH 7.0 by scanning from 0.00 to 1.20 V using a step potential of 5.0 mV and an interval time of 0.50 s (effective scan rate ≈10.1 mV $s^{-1}$); the pulse modulation amplitude and modulation time were set to 50 mV and 50 ms, respectively. For each DPV measurement, a 50 μL aliquot of the test solution was applied to the electrode, covering the integrated three-electrode system. For the independent electrochemical characterization of TNZ, DPV measurements were performed in PBS containing 8.5 μM TNZ over the −1.0 to 0.0 V potential range, using the same pulse parameters and baseline-correction procedure. Calibration measurements were performed using three independently fabricated SPE_C_CNT-HTA-X5 electrodes prepared according to the same modification protocol. For each electrode, DPV measurements were recorded sequentially at CIP concentrations of 4.2, 8.5, 17, and 34 μM, following an ascending concentration order. Between consecutive measurements, each electrode was rinsed using 50 μL of Milli-Q water for 10 min, without UVA irradiation. This procedure was adopted to minimize potential carry-over effects by avoiding exposure to the highest CIP concentration before measurements at lower concentrations. The influence of residual CIP adsorption and the effectiveness of UVA-assisted cleaning were investigated separately in the regeneration experiments, as discussed in Section 3.5.

The DPV oxidation peak currents were extracted independently from each electrode after applying a consistent baseline-correction procedure to all recorded voltammograms, based on the moving minimum method with a 51-point window. At each concentration, mean peak currents and sample standard deviations were calculated from the three individual measurements. The individual calibration slopes were also determined to evaluate sensor-to-sensor reproducibility.

For the regeneration assessment, one of the independently fabricated SPE_C_CNT-HTA-X5 electrodes was subjected to repeated detect–clean cycles using 34 μM CIP, with a DPV measurement in PBS recorded after each 10 min UVA cleaning step. In addition, calibration measurements at 4.2, 8.5, and 17 μM CIP were performed before and after the 10 min UVA treatment in order to evaluate the retention of the analytical response at lower concentrations. The near-neutral pH was intentionally selected for all CIP sensing experiments to provide stable and reproducible electrochemical conditions while avoiding measurements under artificially acidic conditions. Quantification was performed using the ciprofloxacin Faradaic peak occurring in the 0.60-0.90 V region, attributed to the electro-oxidation of CIP at the carbon-based interface (with the corresponding reduction signal being weak/absent under the adopted DPV conditions). A potentiostat/galvanostat Model No. 302N equipped with impedance module (FRA32M, Autolab, Metrohm, The Netherlands) was used. Data acquisition and instrument control were carried out with the dedicated Autolab NOVA 2.1 software. Data analysis (peak extraction, regression fitting, and plotting) was performed using dedicated Python 3.12 scripts, deposited on Zenodo and accessible via the corresponding link. Blank measurements were performed in 0.10 M PBS at pH 7.0 in the absence of CIP. The resulting DPV curves were processed using the same 51-point moving-minimum baseline correction applied to the CIP measurements. Since no CIP oxidation peak is expected in the blank, the maximum residual current within the 0.60–0.90 V potential window was considered as the blank-equivalent peak response. The standard deviation (σ) was calculated from 10 independent blank measurements and used to estimate the LOD and LOQ. All electrochemical measurements were conducted with the selected screen-printed electrode configuration as detailed in Section 2.4, under the experimental conditions specified for each technique. All CIP measurements in this study were deliberately performed at pH 7.0. Photo-cleaning (electrode regeneration) was carried out using an 18 W UVA/blue DULUX OSRAM lamp. The distance between the lamp and the electrode surface was fixed at 6 cm, and the spectral irradiance measured at the electrode position is reported in Figure S1. The integrated UVA irradiance (315–400 nm) was approximately 3.23 mW ${\mathrm{cm}}^{-2}$. To ensure reproducible irradiation conditions, the lamp and electrodes were enclosed in a black, light-shielded chamber throughout the treatment. The distance between the light source and the electrode surface was fixed at 6 cm. During UVA irradiation, the working electrode surface was kept wet using 50 μL of Milli-Q water throughout the 10 min photo-cleaning treatment. Preliminary photo-cleaning tests were performed using irradiation times of 5 and 10 min. After 5 min of UVA exposure, a residual CIP-related oxidation peak was still detectable in the DPV response recorded in CIP-free PBS, whereas after 10 min the residual peak was no longer clearly distinguishable. Therefore, 10 min was selected as the optimal irradiation time under the investigated conditions. For each measurement, a fresh CIP solution at the same concentration was used.

## 3. Results and Discussion

### 3.1. Physical and Chemical Characterization

**Figure 2:**
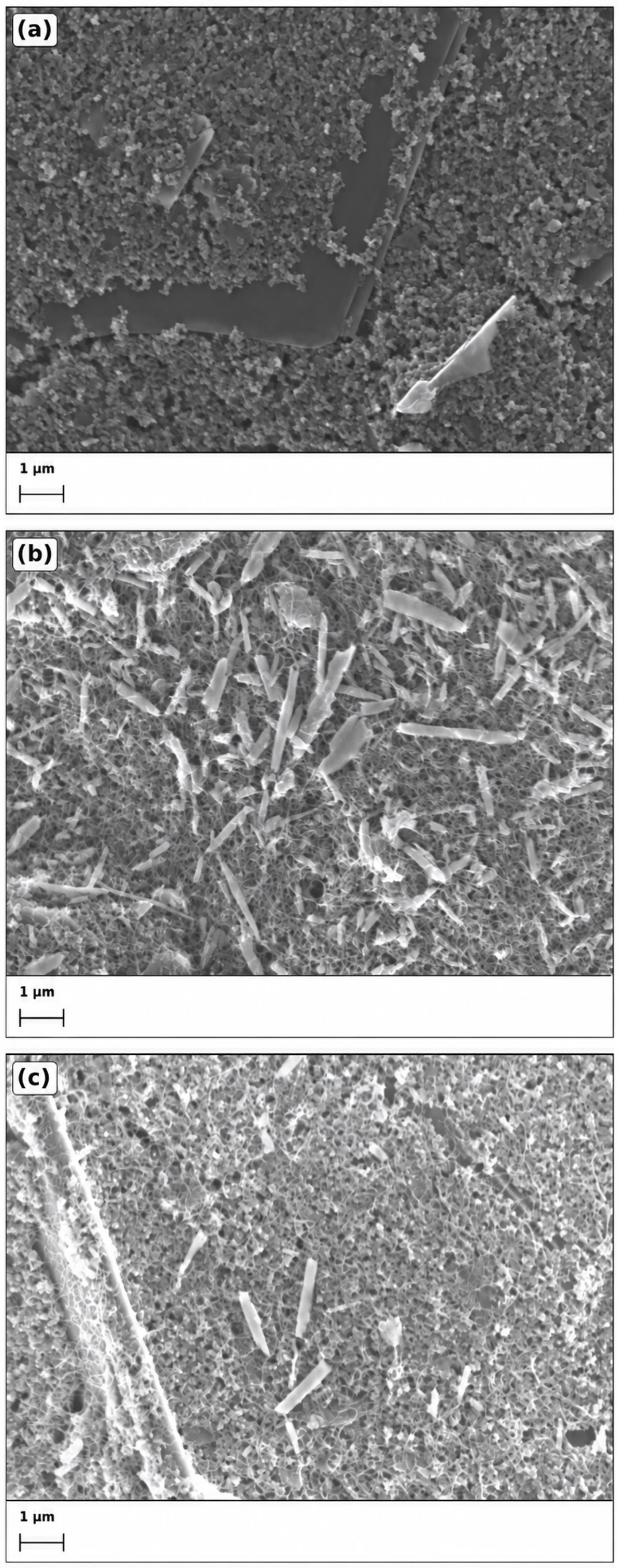
SEM micrographs of the carbon screen-printed electrode before and after modification with CNT–halloysite composites: (**a**) bare SPE_C, (**b**) SPE_C_CNT-HTA, and (**c**) SPE_C_CNT-HTB. Scale bar: 1 μm.

The surface morphology of the bare and modified screen-printed carbon electrodes was investigated by SEM, as shown in Figure 2. The unmodified SPE_C electrode exhibited the typical granular and relatively compact morphology of the carbonaceous screen-printed surface. After modification, both SPE_C_CNT-HTA and SPE_C_CNT-HTB showed a clear change in surface texture, confirming the successful deposition of the CNT–halloysite composite onto the electrode. In both cases, the SEM micrographs revealed the coexistence of an entangled CNT network and halloysite-derived micro/nanostructured features distributed over the carbon substrate, indicating the effective incorporation of both components within the coating. Carbon nanotubes are well known to improve the electrochemical behavior of modified electrodes by increasing the accessible surface area and facilitating electron-transfer processes, while halloysite nanotubes provide a high-surface-area scaffold that is advantageous for the formation of hybrid sensing interfaces [25,26].

A comparison between the two composite coatings indicates that both modified electrodes exhibit a rather uniform surface coverage, without evident macroscopic discontinuities in the deposited layer. The main morphological difference concerns the amount and compactness of the deposited material. In particular, SPE_C_CNT-HTA appears characterized by a more abundant and continuous composite overlayer, whereas SPE_C_CNT-HTB shows a comparatively lower surface loading, while still maintaining a homogeneous distribution over the analyzed area. From an electrochemical perspective, these observations are relevant because the amount of deposited active material can directly influence the available electroactive surface area and the number of accessible charge-transfer sites at the electrode/electrolyte interface [25,27].

**Figure 3:**
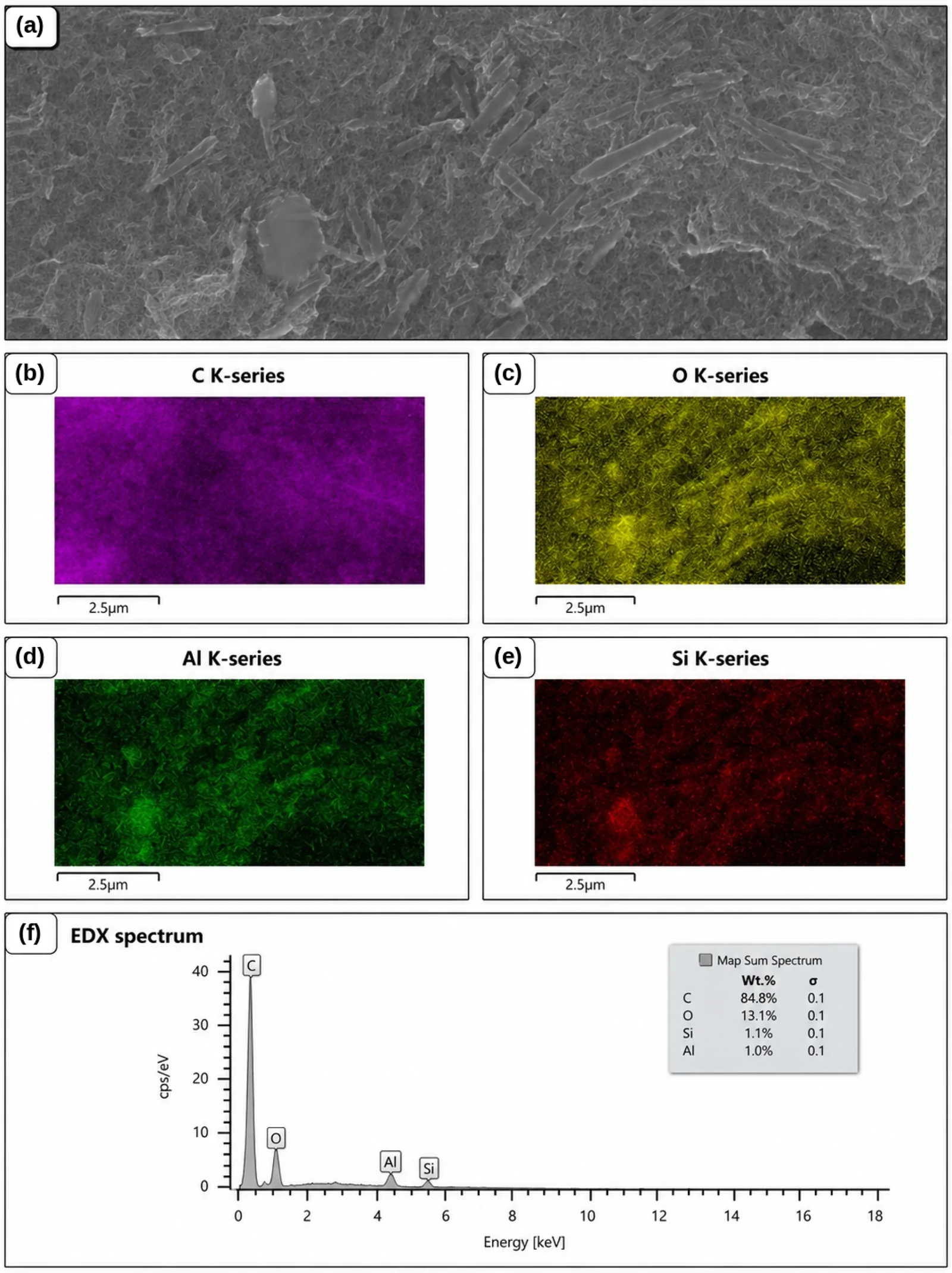
(**a**) SEM view of the analyzed portion of the SPE_C_CNT-HTA electrode surface. EDX elemental mapping of: (**b**) C, (**c**) O, (**d**) Al, (**e**) Si, and (**f**) summed EDX spectrum acquired on the same region. The elemental maps confirm the presence and spatial distribution of carbon nanotubes together with halloysite-derived elements on the modified electrode.

**Figure 4:**
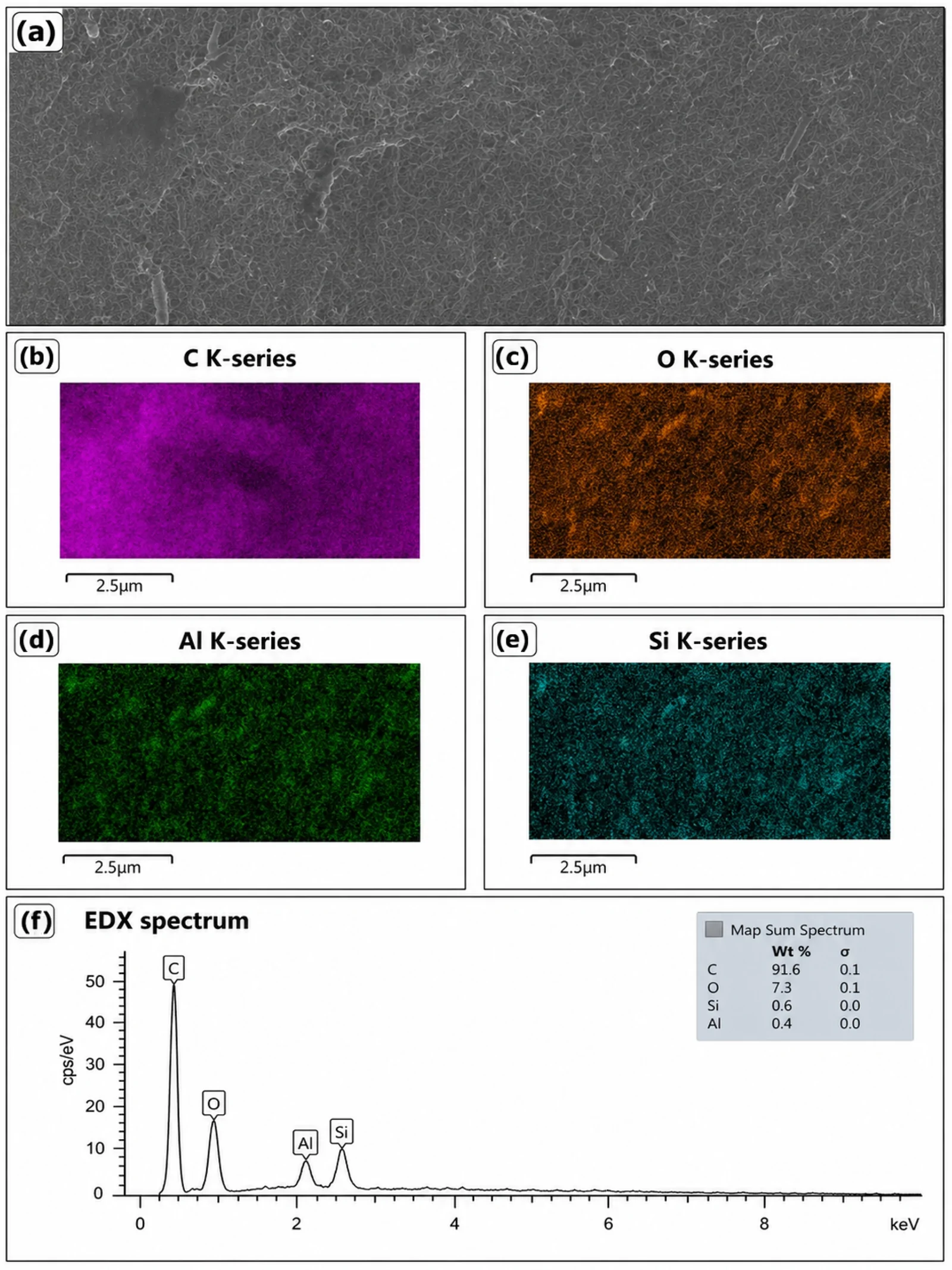
(**a**) SEM view of the analyzed portion of the SPE_C_CNT-HTB electrode surface. EDX elemental mapping of: (**b**) C, (**c**) O, (**d**) Al, (**e**) Si, and (**f**) summed EDX spectrum acquired on the same region. The elemental maps confirm the presence and spatial distribution of carbon nanotubes together with halloysite-derived elements on the modified electrode.

The elemental composition and spatial distribution of the modified layers were further investigated by EDX elemental mapping, as reported in Figure 3 for SPE_C_CNT-HTA and Figure 4 for SPE_C_CNT-HTB. In both samples, the summed EDX spectra and the corresponding elemental maps confirmed the presence of the main constituents of the hybrid coating. The intense C signal is mainly associated with the carbonaceous screen-printed substrate together with the CNT-based overlayer, while the detection of Al and Si is consistent with the aluminosilicate composition of halloysite nanotubes. The O signal is consistent with the presence of the clay-based component within the deposited composite.

EDX analysis confirmed the incorporation of the CNT–halloysite hybrid coating onto the electrode surface. In particular, the Al and Si signals exhibited an approximately 1:1 ratio, in agreement with the expected aluminosilicate composition of halloysite. Although EDX should be regarded as a semi-quantitative technique, the relative intensity of these signals also highlights the different halloysite loading in the two investigated coatings. Specifically, the sample reported in Figure 3f shows more pronounced Al and Si contributions, indicating a higher halloysite content, whereas lower Al and Si signals are observed for the sample shown in Figure 4f, consistent with a reduced amount of halloysite within the coating. Moreover, the corresponding elemental maps reveal a reasonably homogeneous distribution of the detected elements over the analyzed areas, in agreement with the SEM observations.

### 3.2. Electrochemical Characterization

Electrochemical characterization was carried out to compare the interfacial charge-transfer properties of the five investigated electrodes and identify the most suitable configuration for ciprofloxacin sensing. The following platforms were evaluated: SPE_C, SPE_C_CNT, SPE_C_HTA, SPE_C_CNT-HTA, and SPE_C_CNT-HTB. Figure S2 in the Supporting Information reports the Nyquist plots and the corresponding fitting results obtained for all electrodes. In order to enable a direct comparison across the full series, the impedance spectra were analyzed using the same Randles-type equivalent circuit, in which the solution resistance ($R_{s}$) is in series with a parallel branch composed of a constant phase element (CPE) and the charge-transfer resistance ($R_{ct}$), complemented by a Warburg diffusion element (*W*) [28]. Although some modified surfaces may reasonably exhibit additional non-idealities associated with coating heterogeneity and distributed time constants, the selected circuit provided a satisfactory comparative description of the experimental response. The extracted fitting parameters are summarized in Table 2.

Overall, $R_{s}$ values were comparable for all configurations (Table 2), indicating that the solution-related contribution remained essentially unchanged upon surface modification. In contrast, pronounced differences were observed in the fitted $R_{\mathrm{ct}}$ values, suggesting that the surface modification influences the interfacial electrochemical response under the investigated conditions. The bare SPE_C exhibited $R_{ct}=40.5$ kΩ. This relatively high value is not unexpected for a commercial screen-printed carbon electrode, whose interfacial response is governed by the composite nature of the printed carbon ink, including binders/additives and a limited fraction of readily accessible electroactive domains, rather than by the intrinsic conductivity of ideal graphitic carbon alone [29,30]. The SPE_C_CNT electrode showed a slightly higher fitted $R_{\mathrm{ct}}$ (47.3 kΩ), which may be associated with non-uniform CNT coverage and incomplete network connectivity. This interpretation is consistent with the known tendency of CNTs to aggregate and with the dependence of their electrical properties on dispersion quality and network organization [31,32]. The SEM micrograph of the CNT-only coating reported in Figure S3 of the Supporting Information shows a markedly non-uniform morphology, providing qualitative support for this interpretation.

A similar increase in $R_{ct}$ was observed for SPE_C_HTA (47.6 kΩ), in agreement with the intrinsically insulating aluminosilicate nature of halloysite when it is not coupled to a conductive framework [26,33].

Interestingly, the lowest fitted $R_{\mathrm{ct}}$ values were obtained for the hybrid CNT/HT electrodes, suggesting a favorable combined effect of the two components on the interfacial electrochemical response. In particular, SPE_C_CNT-HTA exhibited $R_{\mathrm{ct}}=16.5$ kΩ, corresponding to a reduction of approximately 60% compared with bare SPE_C, while SPE_C_CNT-HTB also showed a lower fitted resistance ($R_{\mathrm{ct}}=27.8$ kΩ). These observations are consistent with a possible complementary role of the two components, in which CNTs may favor electrical connectivity and halloysite may contribute to interfacial accessibility and surface organization [26,34,35]. However, the EIS results alone do not allow the individual contributions of these mechanisms to be directly quantified.

The CPE exponent (*N*) ranged between 0.88 and 0.99 (Table 2), confirming the non-ideal capacitive behavior typically associated with printed carbon electrodes and nanostructured coatings, where surface heterogeneity, roughness, porosity, and distributed charge-transfer environments lead to a deviation from ideal capacitive response [36,37]. CNT-containing interfaces generally exhibited lower *N* values (e.g., 0.88–0.92), consistent with an increased interfacial heterogeneity induced by the nanostructured coating. Finally, the Warburg-related parameter ($Y_{0}$) did not show a monotonic trend across the investigated electrodes (Table 2). Therefore, it was not used as the primary criterion for selecting the optimal sensing interface. Nevertheless, the presence of a low-frequency diffusion contribution in all the fitted spectra indicates that mass-transport processes contribute to the overall interfacial response, as commonly observed for screen-printed and nanostructured electrochemical interfaces [28].

**Figure 5:**
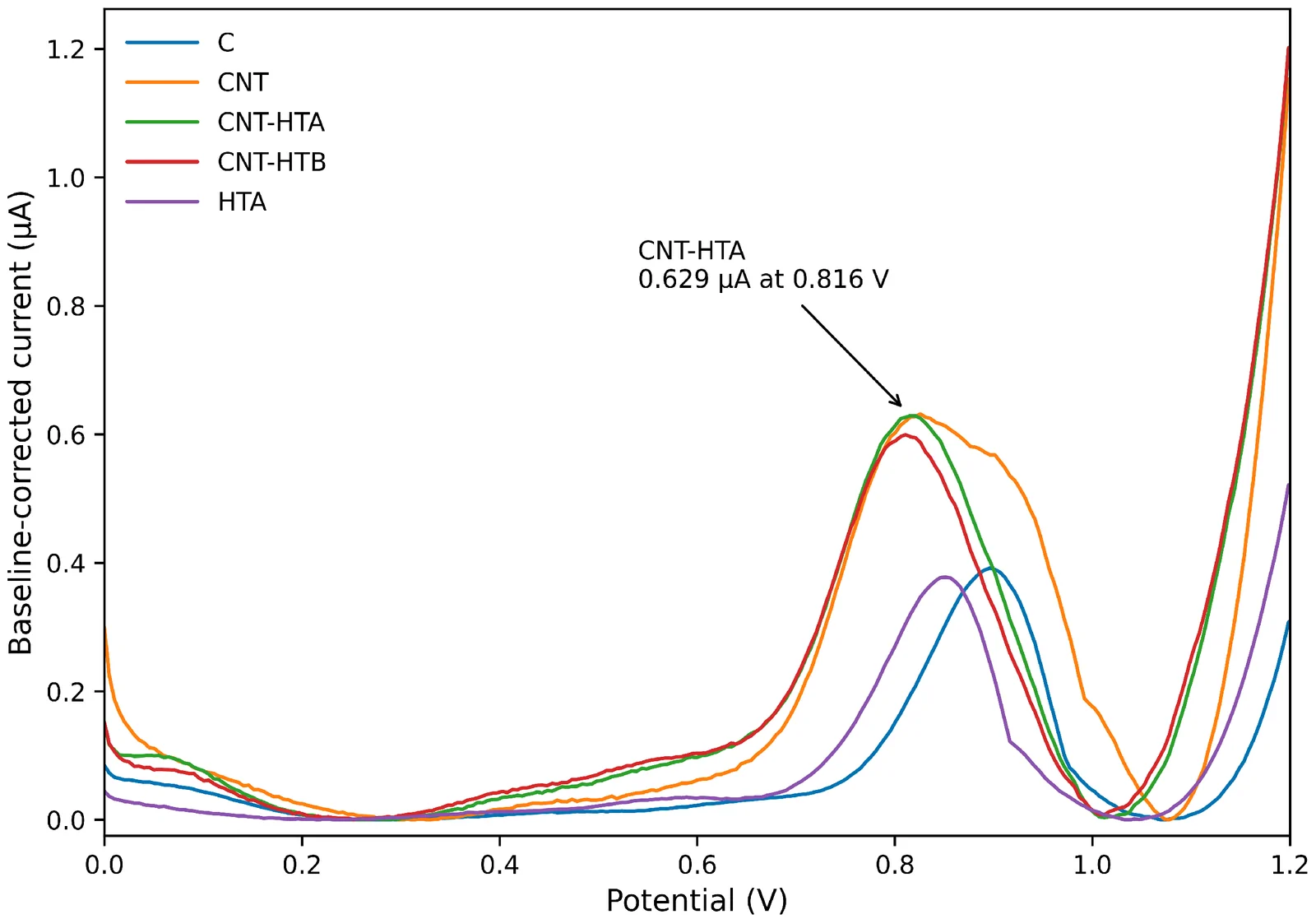
Baseline-corrected DPV responses obtained in PBS containing 34 µM ciprofloxacin for five screen-printed carbon-based electrodes (SPE_C, SPE_C_CNT, SPE_C_CNT-HTA, SPE_C_CNT-HTB and SPE_C_HTA).

To compare the performance of the different electrode configurations, DPV measurements were carried out in PBS containing 34 µM ciprofloxacin (approximately 11–13 µg ${\mathrm{mL}}^{-1}$), i.e., the highest analyte concentration examined in this study [5]. The corresponding baseline-corrected voltammograms are reported in Figure 5. For all samples, a well-defined oxidation peak was observed in the high-potential region, in agreement with the literature [38,39,40,41], although with markedly different intensities depending on the electrode surface modification. The exact position depends on the electrode and the pH of the solution. The observed trend demonstrates that the nature of the modifying layer strongly affects the electrochemical response toward ciprofloxacin. In particular, SPE_C_CNT-HTA provided the highest peak current among all the tested platforms, revealing the most efficient signal detection under the selected experimental conditions. The SPE_C_CNT sample also shows a current peak whose intensity is comparable to that of the SPE_C_CNT-HTA sample. However, for the CNTs alone, the peak appears much broader and the presence of a second contribution is evident, attributable to the non-uniformity of the electrode (as discussed previously and shown in Figure S3).

Based on the initial EIS and DPV characterization, SPE_C_CNT-HTA was identified as the most promising composite composition, combining relatively low charge-transfer resistance with a well-defined CIP oxidation response. The subsequent optimization of modifier loading was performed primarily according to the analytical response toward CIP.

The observed performance may be associated with the complementary properties of CNTs and HT within the hybrid architecture. CNTs may contribute to electrical connectivity and the availability of electroactive surface sites, while halloysite may provide additional adsorption sites that favor local analyte accumulation and influence interfacial accessibility. On this basis, SPE_C_CNT-HTA was selected for subsequent ciprofloxacin-detection experiments.

### 3.3. Analytical Performance and Sensitivity

Based on the morphological and electrochemical characterization discussed above (SEM, EIS, and DPV), the SPE_C_CNT-HTA configuration emerged as the most promising sensing interface, providing the most favorable balance between interfacial charge-transfer properties and electroactive behavior. To further enhance the analytical response toward ciprofloxacin, the modifier loading was systematically increased by repeating the same drop-casting/vacuum-drying step for three and five consecutive depositions, hereafter denoted as X3 and X5, respectively.

The optimization of the deposited CNT–HTA amount revealed a substantial enhancement in the electrochemical response with increasing modifier loading. At a fixed CIP concentration of 34 μM, increasing the number of deposited drops from one (X1) to five (X5) resulted in an approximately 20-fold increase in the oxidation peak current, from about 0.63 to 13.4 μA. This remarkable improvement supported the selection of SPE_C_CNT-HTA-X5 for subsequent calibration and analytical evaluation. Further deposition beyond X5 was not investigated, as excessively thick or compact modifier layers may become counterproductive by increasing the capacitive background and ohmic resistance while hindering analyte diffusion toward the underlying conductive surface, potentially compromising the Faradaic-to-background ratio and analytical sensitivity [42,43]. The corresponding EIS characterization, including the investigated multilayer configurations, is reported in the Supporting Information (Figure S4). In parallel, DPV measurements were performed at a fixed CIP concentration of 34 μM for the different electrode modifications. This concentration, already adopted during the preliminary screening stage, was intentionally retained as a common benchmark to enable a direct comparison of their electroanalytical responses under identical conditions. The corresponding DPV responses are reported in the Supporting Information (Figure S5).

Although SPE_C_CNT-HTA-X5 exhibited a higher apparent charge-transfer resistance (32.0 kΩ) than some of the other configurations, including SPE_C_CNT-HTB-X3 (15.0 kΩ), electrode selection was based primarily on the analytical DPV response toward CIP. The EIS-derived resistance describes the response to the ferricyanide redox probe and does not necessarily correlate directly with CIP oxidation currents, which may also depend on analyte adsorption, interfacial accessibility, and the deposited modifier loading.

**Figure 6:**
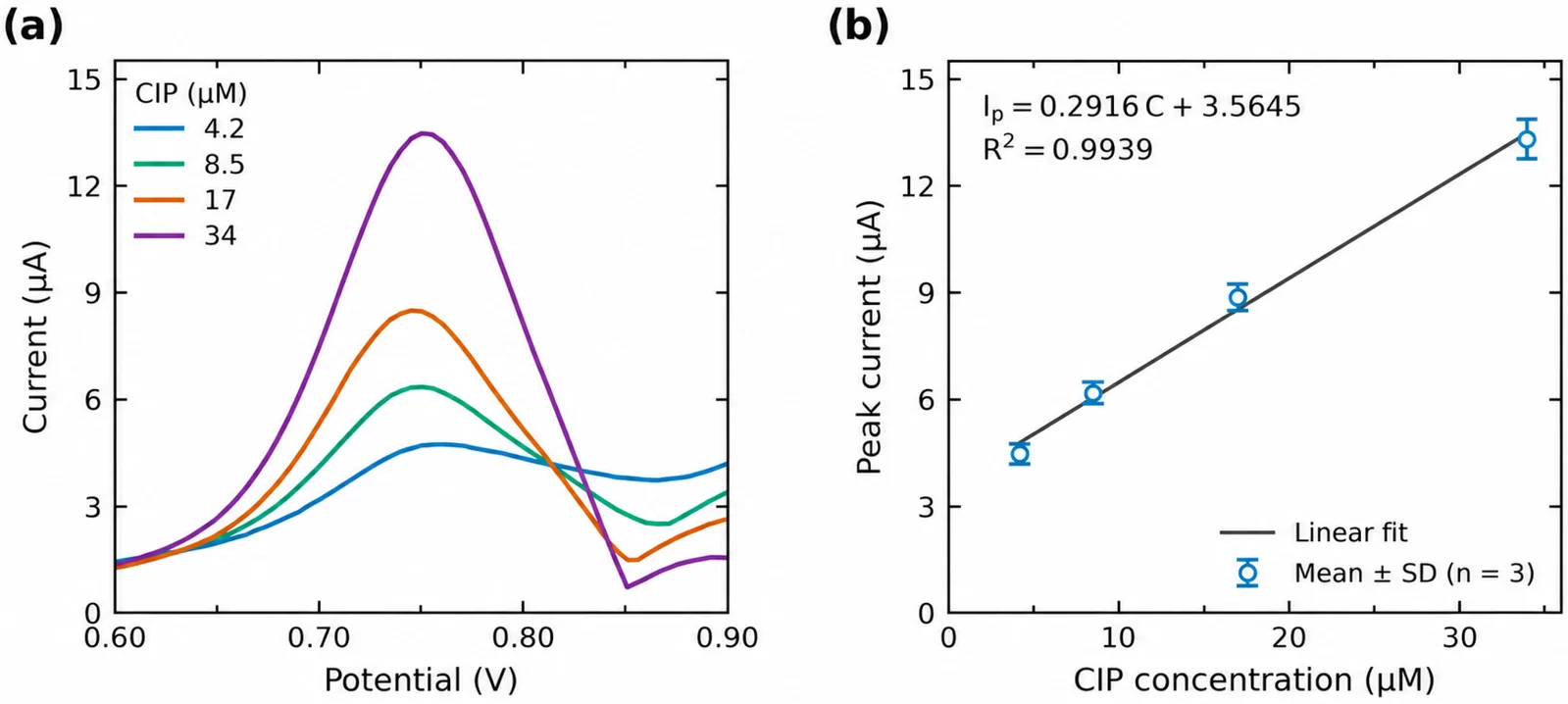
Electrochemical response of SPE_C_CNT-HTA-X5 electrodes toward CIP. (**a**) Representative baseline-corrected DPV curves recorded using a sensor at CIP concentrations of 4.2, 8.5, 17, and 34 μM. (**b**) Calibration curve obtained from the mean oxidation peak currents independently extracted from three fabricated electrodes at each CIP concentration; error bars represent ±1 sample standard deviation (n=3). Baseline correction was performed individually for each DPV trace using a centered 51-point moving minimum. The solid line represents an unweighted linear fit with a free intercept over the investigated 4.2–34 μM concentration interval. No smoothing or potential-axis alignment was applied.

Figure 6a shows representative baseline-corrected DPV curves recorded using one of the three independently fabricated SPE_C_CNT-HTA-X5 electrodes at CIP concentrations of 4.2, 8.5, 17, and 34 μM. A progressive increase in the oxidation peak current was observed with increasing CIP concentration, demonstrating the concentration-dependent electrochemical response of the sensing interface. The calibration results obtained from all three independently fabricated electrodes are presented in Figure 6b, allowing the evaluation of sensor-to-sensor reproducibility.

Figure 6b reports the calibration curve obtained by plotting the mean DPV oxidation peak current as a function of CIP concentration for the three independently fabricated electrodes. A linear relationship was observed over the investigated concentration range (4.2–34μM), described by the following calibration equation:

(1) $$I_{p}\;\left(\mu A\right)=0.2916\;C_{\mathrm{CIP}}\;\left(\mu M\right)+3.5645\;$$

where $I_{p}$ is the baseline-corrected oxidation peak current and $C_{\mathrm{CIP}}$ is the ciprofloxacin concentration. The corresponding correlation coefficient was $R^{2}=0.9939$.

The individual electrode sensitivities ranged from 0.2814 to $0.3056\;\mu A\;\mu M^{-1}$, yielding a mean sensitivity of $\left(0.2916\pm 0.0125\right)\;\mu A\;\mu M^{-1}$, where the uncertainty represents the sample standard deviation of the three individual calibration slopes. The resulting relative standard deviation (RSD) was 4.29%, indicating good sensor-to-sensor reproducibility.

The error bars in Figure 6b represent the sample standard deviations of the peak currents measured using the three independent electrodes at each concentration. The observed analytical response is consistent with the complementary contributions of the CNT network, which provides conductive pathways, and halloysite, which may facilitate local CIP adsorption through surface interactions.

Considering the geometric working-electrode area of $0.126\;{\mathrm{cm}}^{2}$, the mean area-normalized sensitivity was approximately $2.32\;\mu A\;\mu M^{-1}\;{\mathrm{cm}}^{-2}$.

In order to evaluate the analytical performance of the SPE_C_CNT-HTA-X5 sensor, blank measurements were carried out under the same experimental conditions in the absence of the target antibiotic. The standard deviation of the blank current signal (σ) was calculated from multiple measurements and used for the estimation of LOD and limit of quantification (LOQ) [44].

The calibration curve parameters, obtained from linear regression of the DPV peak current versus antibiotic concentration, are summarized in Table 3. Good linearity was observed, as confirmed by the high correlation coefficient.

The reported calibration slope, intercept, and correlation coefficient were obtained by linear regression of the mean peak currents. The standard deviation and relative standard deviation of the sensitivities were calculated from the slopes of the three individual electrode calibrations.

Based on the standard deviation of the blank signal ($\sigma =3.272\times 10^{-9}\;A$), obtained from 10 independent blank measurements, and the mean calibration slope ($m=2.916\times 10^{-7}\;A\;\mu M^{-1}$), the LOD and LOQ were calculated using the conventional 3σ/m and 10σ/m criteria, respectively. The resulting values were approximately 0.034μM (34nM) for the LOD and 0.112μM (112nM) for the LOQ, as summarized in Table 4. These values represent calculated analytical detection and quantification limits, estimated using the conventional 3σ/m and 10σ/m criteria, respectively, based on the blank signal variability and the calibration sensitivity [45,46]. Since the experimental calibration was performed over the 4.2–34 μM concentration range, the calculated LOD and LOQ should be regarded as analytical estimates rather than experimentally verified thresholds at nanomolar concentrations.

To contextualize the analytical figures of merit of the proposed SPE_C_CNT-HTA-X5 sensor, the calculated LOD of 0.034μM (34nM) and LOQ of 0.112μM (112nM), estimated from the blank response using 3σ/m and 10σ/m, respectively, were compared with representative electrochemical platforms for ciprofloxacin detection reported in the literature. As summarized in Table 5, simpler electrode development approaches achieve LODs in the micromolar range. More complex approaches that leverage biological recognition (e.g., aptamers) and/or molecularly imprinted architectures, often combined with noble-metal nanostructures and/or more elaborate fabrication steps, achieve LODs in the nanomolar range [13,14,15].

The area-normalized sensitivity was calculated by dividing the calibration slope by the geometric working-electrode area ($A=0.126\;{\mathrm{cm}}^{2}$, corresponding to a diameter of 4mm).

The comparison in Table 5 highlights the design trade-off that motivated the present work. Several sophisticated CIP sensors reach lower detection limits, particularly when molecular recognition elements, noble-metal nanostructures, or multistep electrode modifications are employed. In contrast, the present strategy prioritizes a simple drop-casting modification of commercial screen-printed carbon electrodes using fully aqueous CNT/HT dispersions and couples this preparation route with post-measurement regeneration (as discussed in Section 3.5). Within this design space, the calculated LOD of 34nM and the mean area-normalized sensitivity of approximately $2.32\;\mu A\;\mu M^{-1}\;{\mathrm{cm}}^{-2}$ support the analytical performance of the proposed sensor. These results should be considered together with the relatively simple aqueous fabrication procedure and the possibility of partial electrode regeneration and reuse. It should be noted that Table 5 provides a comparison of analytical performance rather than a quantitative environmental assessment. Although the proposed approach avoids organic solvents during modifier preparation and demonstrates preliminary electrode reuse, its overall environmental impact, including the energy requirements associated with sonication, vacuum drying, and UVA treatment, has not been quantified.

The incorporation of HT may provide adsorption sites that facilitate CIP adsorption, while the CNT network maintains efficient charge-transfer pathways. In fact, HT contains a high density of OH groups, which may play a dual role in ciprofloxacin detection: (i) they modulate the surface charge of the working electrode, thereby promoting antibiotic adsorption; and (ii) they may be directly involved in the oxidation of ciprofloxacin [50]. Although these findings support the potential contribution of halloysite to the sensing response, the specific accumulation mechanism remains to be established. The improved response of CNT-HTA-X5 is consistent with a favorable balance between these contributions. However, CIP adsorption may involve multiple interactions, including electrostatic attraction and surface complexation with Al–OH groups [24,51], which could also contribute to persistent electrode fouling. Further mechanistic investigations, including scan-rate and adsorption-time dependence, would be required to distinguish and quantify these contributions.

The pH used throughout the calibration is also relevant to this interpretation. At pH 7.0, CIP is predominantly zwitterionic [22], and the measurement is performed without deliberately shifting the analyte toward its more cationic form. Although mild acidification could increase the cationic CIP fraction and potentially enhance electrostatic interactions, the predominantly zwitterionic nature of CIP at pH 7.0 suggests that electrostatic attraction alone is unlikely to fully explain its interaction with the sensing surface. We intentionally retained pH 7.0 to evaluate the sensor under controlled, near-neutral conditions rather than under acid-optimized conditions. However, the use of PBS does not fully reproduce the chemical complexity of natural waters. Therefore, the reported analytical figures of merit should be interpreted as performance under controlled laboratory conditions.

### 3.4. Effect of Tinidazole on the CIP Response

**Figure 7:**
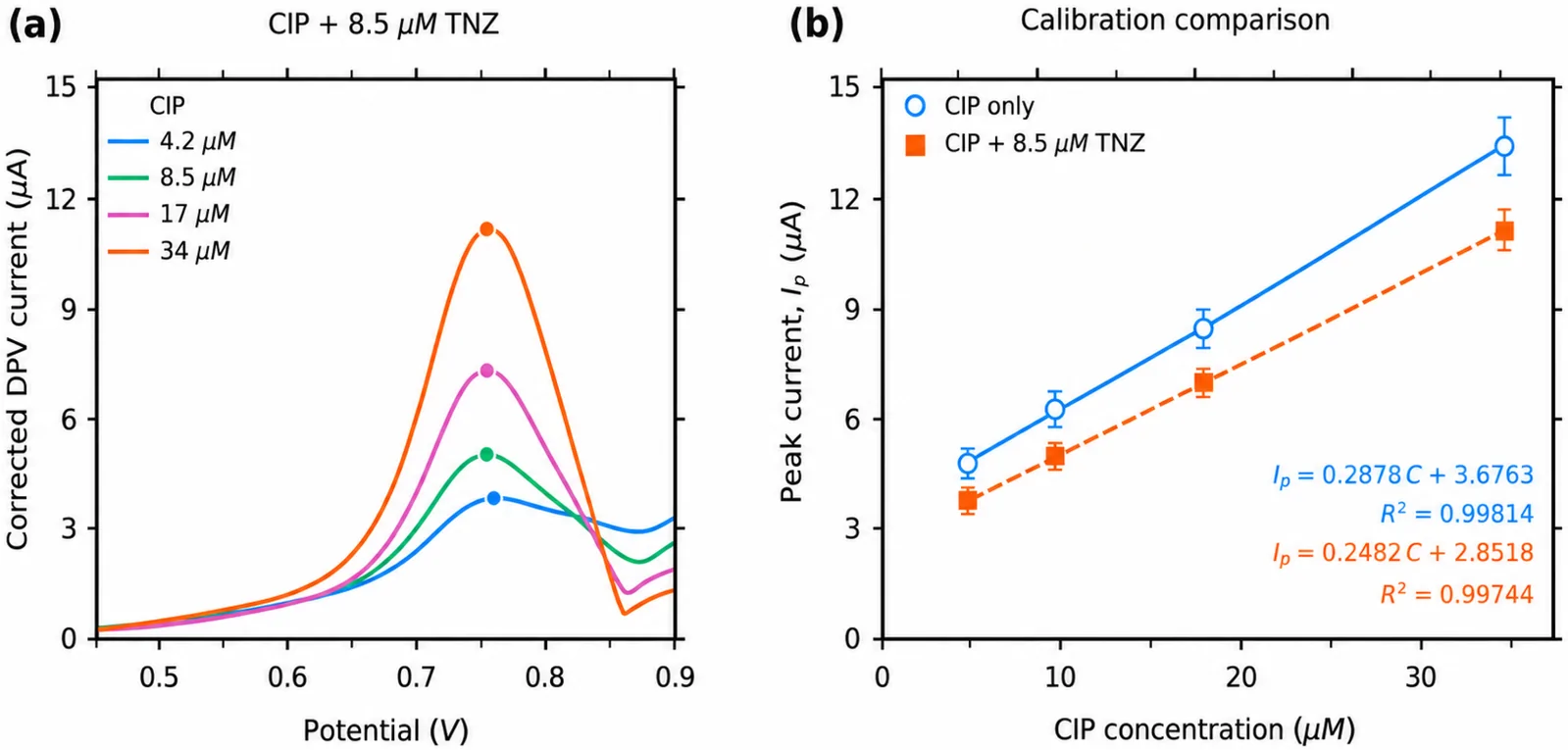
Effect of tinidazole (TNZ) on the ciprofloxacin (CIP) response of the SPE_C_CNT-HTA-X5 electrode. (**a**) Baseline-corrected DPV curves at CIP concentrations of 4.2, 8.5, 17, and 34 μM in the presence of 8.5 μM TNZ; circles identify the extracted peak maxima. The displayed potential interval is 0.45–0.90 V versus the integrated Ag pseudo-reference. (**b**) Comparison of the calibrations obtained using the same electrode without TNZ (open circles, solid line) and with 8.5 μM TNZ (filled squares, dashed line). Symbols represent individual measurements, and lines are unweighted linear fits with free intercepts over 4.2–34 μM. Both datasets were corrected using a centered 51-point moving minimum without smoothing. In the fitted equations, $I_{p}$ and *C* are expressed in μA and μM, respectively. Error bars represent an indicative relative uncertainty of ±4% for both CIP and CIP/TNZ measurements, based on the experimentally observations.

Tinidazole (TNZ), a nitroimidazole antimicrobial, was selected as a model interfering compound because it is also used in combined pharmaceutical formulations with ciprofloxacin [52]. An independent DPV measurement of 8.5 μM TNZ (Figure S8) revealed a main reduction peak at approximately −0.65 V versus the integrated Ag pseudo-reference, well separated from the CIP oxidation peak observed at approximately +0.74–0.81 V. This electrochemical distinction, together with the pharmaceutical relevance of TNZ, supported its selection for the interference study, allowing the influence of TNZ on the CIP response to be investigated without direct overlap between their characteristic peaks. Accordingly, DPV measurements were performed using SPE_C_CNT-HTA-X5 electrodes at CIP concentrations of 4.2, 8.5, 17, and 34 μM, both in the absence and in the presence of a fixed TNZ concentration of 8.5 μM. Both datasets were processed using the same centered 51-point moving-minimum baseline correction, without smoothing. Peak currents were extracted as the largest local maxima within the 0.60–0.90 V interval versus the integrated Ag pseudo-reference. Figure 7a shows the baseline-corrected DPV curves recorded in the presence of TNZ, while Figure 7b compares the corresponding calibration with that obtained for CIP alone.

Unweighted linear regression with a free intercept yielded slopes of 0.2878±0.0088 and 0.2482±0.0089 μA μ$M^{-1}$ for CIP alone and CIP with TNZ, respectively. The corresponding intercepts were 3.676±0.172 and 2.852±0.174 μA, with $R^{2}$ values of 0.99814 and 0.99744. The reported uncertainties are the standard errors of the regression coefficients. The slope obtained in the presence of TNZ was 13.8% lower than that obtained for CIP alone; individual peak currents were also lower by 14.0–21.8% across the investigated concentration range. Thus, a linear concentration-dependent response was retained in the tested CIP/TNZ mixture, although the calibration obtained for CIP alone cannot be assumed to apply unchanged. These results provide a preliminary assessment of the CIP response in the presence of TNZ, rather than a comprehensive validation of selectivity.

### 3.5. Light-Assisted Regeneration and Proof-of-Concept Reuse

**Figure 8:**
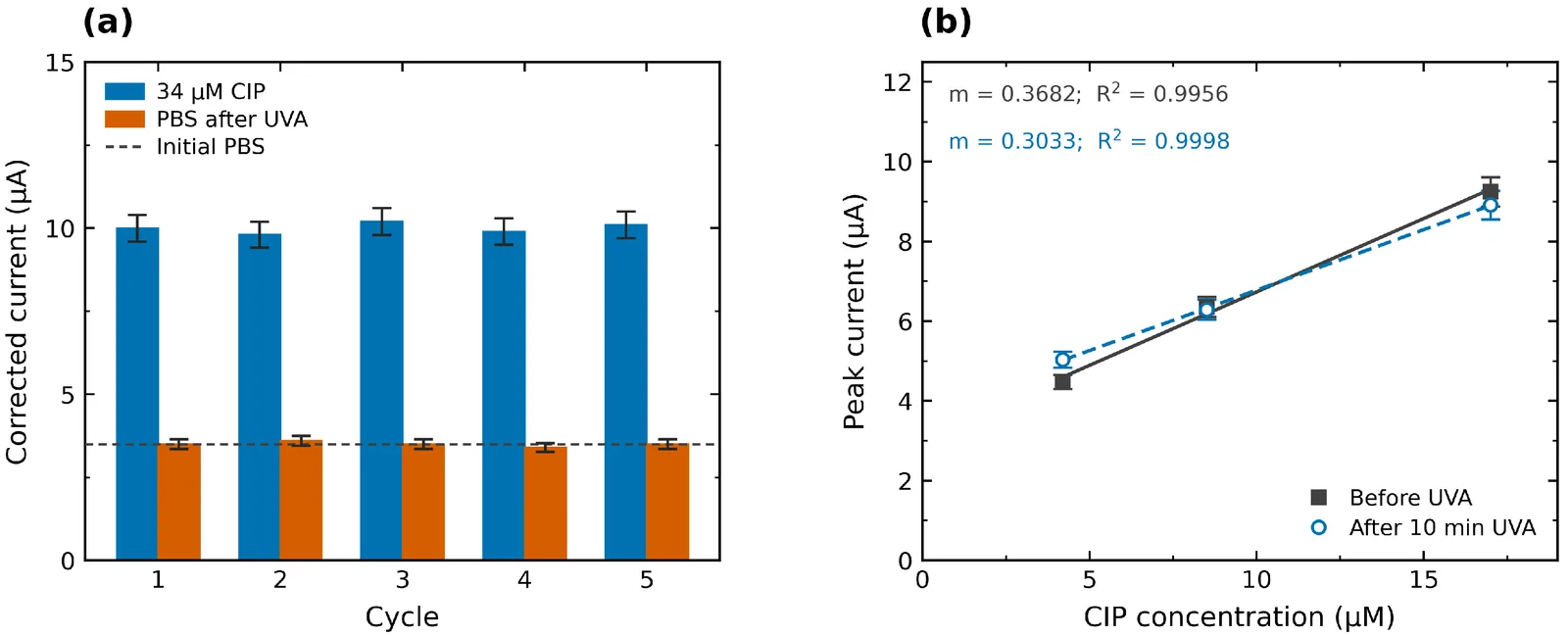
UVA-assisted regeneration and reuse of the SPE_C_CNT-HTA-X5 electrode. (**a**) Corrected current values measured during five consecutive detect–clean cycles performed using 34 μM CIP. After each detection step, the electrode was cleaned for 10 min under UVA and then tested in PBS; the dashed line indicates the initial PBS response of the fresh electrode. (**b**) Calibration plots obtained before and after 10 min UVA treatment using the lower CIP concentrations of 4.2, 8.5, and 17 μM, showing partial retention of sensitivity after regeneration.

The possibility of regenerating the SPE_C_CNT-HTA-X5 electrode was investigated by comparing the effects of water rinsing and UVA-assisted cleaning on the sensing interface. Figure 8 summarizes the regeneration behavior of the SPE_C_CNT-HTA-X5 electrode after CIP detection. Figure S9 in Supplementary File compares the uncorrected DPV responses recorded in CIP-free PBS after water rinsing alone and after a subsequent 10 min UVA treatment. After water rinsing, a residual oxidation feature is still visible at approximately 0.8–0.9 V, indicating incomplete removal of the CIP-related response. After UVA treatment, this residual feature is no longer clearly resolved, supporting the effectiveness of the light-assisted cleaning procedure. It is worth noting that the residual CIP-related feature observed after water rinsing was recorded following exposure to 34 μM CIP, corresponding to the highest concentration investigated. At lower concentrations (4.2–17 μM), residual CIP adsorption is expected to be less pronounced. This observation reinforces the rationale for performing the calibration measurements in ascending concentration order, with intermediate water rinsing, thereby avoiding exposure to the highest CIP concentration before the lower-concentration measurements. The good linearity and sensor-to-sensor reproducibility reported in Section 3.3 are consistent with the effectiveness of this procedure in obtaining reproducible calibration responses. To further evaluate the repeatability of the regeneration step, five consecutive detect–clean cycles were performed using 34 μM CIP, and the corrected current measured in PBS after each UVA treatment was compared with the initial PBS value (Figure 8a). In all cycles, the PBS signal recorded after the 10 min UVA treatment remained very close to the initial PBS response of the fresh electrode, indicating reproducible baseline recovery after repeated cleaning steps.

Figure 8b compares the calibration curves obtained using the same electrode before and after UVA treatment. To assess whether the regenerated electrode also preserved its analytical response at lower concentrations, calibration measurements were performed before and after a 10 min UVA treatment using CIP concentrations of 4.2, 8.5, and 17 μM (Figure 8b). No significant change is observed in the investigated range, with the peak currents being within the error bars. However, to extrapolate the behavior at lower and higher concentrations the sensitivity was calculated before and after UVA treatment. The initial slope of the linear fit was 0.3682 μA μ$M^{-1}$ with $R^{2}$ = 0.9956, whereas after UVA treatment the slope was 0.3033 μA μ$M^{-1}$ with $R^{2}$ = 0.9998. The regenerated electrode therefore retained approximately 82.4% of its initial sensitivity, corresponding to a decrease of approximately 17.6%.

These results demonstrate that UVA-assisted cleaning allows subsequent CIP detection using the same electrode, although the initial analytical performance is not fully restored. The decrease in sensitivity after regeneration suggests that the cleaning procedure may not completely recover the original sensing interface. Nevertheless, the persistence of a concentration-dependent response after UVA treatment supports the feasibility of partial electrode regeneration and reuse.

Overall, these results should be interpreted as a proof-of-concept demonstration of electrode regeneration and partial reuse. The repeated recovery of the PBS baseline after five successive detect–clean cycles, together with the retained calibration response at lower CIP concentrations after UVA treatment, supports the effectiveness of the proposed light-assisted cleaning approach. Further experiments involving additional independently prepared electrodes and more extended cycling tests will nevertheless be required to fully assess the long-term reproducibility and durability of the regenerated sensing interface.

The observed recovery is attributed to a light-assisted regeneration process rather than to an established photocatalytic mechanism. CIP is known to be photosensitive and susceptible to direct photodegradation under UV irradiation [53]. Therefore, the adsorption of CIP onto HT may promote its light-induced transformation, although the present experiments do not allow this contribution to be quantitatively distinguished from adsorption and desorption processes. To investigate this effect, a parallel UV–Vis experiment was performed using 7 mL of a 34 μM CIP solution placed in two separate beakers, one containing CIP alone and the other containing HT at a concentration of 0.125 mg ${\mathrm{mL}}^{-1}$, both exposed to identical UVA irradiation conditions. UV–Vis spectra recorded before and after 10 min of UVA irradiation showed a decrease of approximately 0.5% in the characteristic CIP absorption band centered at 276 nm for CIP alone, whereas a larger apparent decrease of approximately 6.6% was observed in the presence of HT (Figure S6a,b). Importantly, HT was not removed from the suspension before spectral acquisition, and both spectra were recorded in its presence. The optical background in the 340–600 nm region remained comparable before and after irradiation, although no independent HT-only background subtraction was performed. Therefore, the observed 6.6% decrease should be interpreted a change in the CIP-related absorbance due to different effects including adsorption and photo-assisted degradation. These results provide complementary evidence of changes in the CIP/HT system upon UVA exposure, while the electrochemical measurements provide the primary evidence supporting partial electrode regeneration. Complementary SEM micrographs of the optimized SPE_C_CNT-HTA-X5 electrode before use, after CIP sensing, and following UVA-assisted cleaning are reported in Figure S7. The three conditions exhibit comparable surface morphologies, with no evident major structural alterations or significant disruption of the CNT/HT composite coating. These observations suggest that the sensing layer largely preserves its morphological integrity during the sensing and regeneration processes, supporting the feasibility of electrode reuse.

## 4. Conclusions

A reusable electrochemical platform for CIP detection was developed by modifying commercial screen-printed carbon electrodes with a CNT/halloysite hybrid layer prepared exclusively from aqueous dispersions. The two components are proposed to play complementary roles: CNTs provide conductive pathways and electroactive surface area, while halloysite offers potential adsorption sites that may facilitate CIP adsorption through different surface interactions. Among the investigated configurations, SPE_C_CNT-HTA-X5 provided the most favorable balance, achieving a mean sensitivity of 0.2916 μA μ$M^{-1}$ over the experimentally investigated 4.2–34 μM calibration range. The estimated LOD of approximately 34 nM was calculated from the blank signal variability and calibration sensitivity, rather than experimentally validated at nanomolar concentrations. Measurements performed using three independently fabricated electrodes demonstrated good sensor-to-sensor reproducibility, with an RSD of 4.29% for the calibration sensitivities.

The significance of the platform lies in this combination of competitive analytical performance and reduced process complexity rather than in pursuing the lowest LOD reported for CIP. No organic solvent is required to prepare or deposit the modifier, and all CIP sensing measurements were intentionally performed in PBS at pH 7.0, ensuring reproducible near-neutral conditions. Although mildly acidic conditions could potentially favor CIP adsorption, the selected pH provides a conservative assessment of the sensor performance. While the results demonstrate the potential of the proposed sensing platform, further investigations involving matrix effects and selectivity, will be necessary to extend the applicability to real water monitoring.

A second distinguishing feature is the recovery of the sensing response by a short UVA-assisted cleaning treatment. In the proof-of-concept reuse experiment, a 10min UVA-assisted treatment reduced the residual CIP-related signal and allowed subsequent measurements using the same electrode. After UVA treatment, the electrode retained approximately 82.4% of its initial sensitivity over the investigated lower concentration interval (4.2–17 μM). Furthermore, five consecutive detect–clean cycles showed that the PBS response after each UVA treatment remained close to the initial baseline value, supporting the repeatability of the light-assisted cleaning process. The available data support a light-assisted removal/transformation of adsorbed CIP, although they do not yet establish a purely photocatalytic mechanism. Taken together, aqueous modifier preparation and preliminary light-assisted electrode reuse offer potential advantages in reducing organic solvent consumption and electrode replacement. However, a quantitative assessment of the overall environmental impact, including energy consumption and electrode durability over extended regeneration cycles, remains necessary.

These results identify CNT/halloysite-modified screen-printed electrodes as a promising low-impact sensing strategy for CIP and provide a design concept that could be extended to other emerging contaminants for which adsorption, simple fabrication, and electrode regeneration are advantageous.

## Figures and Tables

**Table 1:** Summary of the fabrication protocols and electrode codes used in this work. All modifications were carried out by drop-casting aqueous dispersions onto the working electrode area of commercial carbon screen-printed electrodes (SPE_C). Each deposition consisted of 10 μL followed by vacuum drying; multilayer configurations were obtained by repeating the same deposition/drying step.

Electrode	Modifier	# Drops	Volume	Concentration
		(X)	(μL)	($mg/{mL}^{-1}$)
ine SPE_C	None (bare carbon SPE)	0	0	-
SPE_C_HTA	Halloysite nanotubes (HTA)	1	10	0.025
SPE_C_CNT	CNT	1	10	0.03
SPE_C_CNT-HTA	CNT + HTA	1	10	CNT: 0.030; HT: 0.025
SPE_C_CNT-HTB	CNT + HTB	1	10	CNT: 0.030; HT: 0.005
SPE_C_CNT-X3	CNT	3	30	0.03
SPE_C_CNT-HTA-X3	CNT + HTA	3	30	CNT: 0.030; HT: 0.025
SPE_C_CNT-HTB-X3	CNT + HTB	3	30	CNT: 0.030; HT: 0.005
SPE_C_CNT-X5	CNT	5	50	0.03
SPE_C_CNT-HTA-X5	CNT + HTA	5	50	CNT: 0.030; HT: 0.025
SPE_C_CNT-HTB-X5	CNT + HTB	5	50	CNT: 0.030; HT: 0.005

**Table 2:** Electrochemical parameters of the investigated sensors.

Sensors	*Y*_0_(*W*) (μS $s^{1/2}$)	*N*	*Y*_0_(*CPE*) (μS $s^{N}$)	$R_{s}$ (Ω)	$R_{\mathrm{ct}}$ (kΩ)
SPE_C	36.3	0.970	1.24	248	40.5
SPE_C_CNT	30.0	0.882	9.87	253	47.3
SPE_C_HTA	34.9	0.966	1.09	240	47.6
SPE_C_CNT-HTA	33.7	0.928	6.24	228	16.5
SPE_C_CNT-HTB	30.4	0.901	7.12	244	27.8

**Table 3:** Calibration parameters obtained from the mean DPV oxidation peak currents of three independently fabricated SPE_C_CNT-HTA-X5 electrodes.

Parameter	Value
Mean slope, *m* ($A\;\mu M^{-1}$)	$2.916\times 10^{-7}$
Intercept, *q* (A)	$3.5645\times 10^{-6}$
Correlation coefficient, $R^{2}$	0.9939
SD of individual slopes ($A\;\mu M^{-1}$)	$1.25\times 10^{-8}$
RSD of individual slopes (%)	4.29
Area-normalized sensitivity ($\mu A\;\mu M^{-1}\;{\mathrm{cm}}^{-2}$)	2.32
Number of independent electrodes	3

**Table 4:** Analytical figures of merit for the SPE_C_CNT-HTA-X5 sensor, calculated using the mean calibration slope of three independent electrodes.

Parameter	Value
Standard deviation of blank, σ (A)	$3.272\times 10^{-9}$
Limit of detection, LOD (μM)	0.034
Limit of quantification, LOQ (μM)	0.112

**Table 5:** Comparison of the proposed CNT-HTA-X5 sensor with selected electrochemical sensors for ciprofloxacin (CIP). LOD/LOQ values are reported as stated in the corresponding references; unit conversions to µM are provided for direct comparison.

Platform / Electrode	Recognition / Modifier	Tech.	pH	Linear range (µM)	LOD (µM)	LOQ (µM)	Ref.
SPE_C_CNT-HTA-X5 /							
SPE_C (carbon)	CNT + halloysite (aqueous, drop-cast; multilayer X5)	DPV	7	4.2–34	0.034 (34 nM)	0.112	This work
eCNF/CNT/NiCo-GCE	Hierarchical carbon nanocomposite with NiCo nanoparticles	DPV	6.8	0.025–0.30	0.006 (6 nM)	n.r.	[11]
PET-SPCE/AuNPs/							
Aptamer	Printed aptasensor with AuNPs	DPV	7	0.010–0.050	0.003 (3 nM)	0.010 (10 nM)	[13]
EGr/GC	Electrochemically exfoliated graphene on GC	CA	6	0.0033–25	0.001 (1 nM)	0.0033 (3.3 nM)	[12]
Ch-AuMIP/GCE	Chitosan-AuNP molecularly imprinted polymer (MIP)	Voltammetry	7.4	1–100	0.21 (210 nM)	n.r.	[14]
CZF-CPE	Cu-Zn ferrite nanoparticles on carbon paste electrode	AdSV-DPV	3	0.909–4700	0.00258 (2.58 nM)	n.r.	[15]
Pt-RGO/GCE	Platinum-nanoparticle-decorated reduced graphene oxide	DPV	7	10–25	1.53	n.r.	[47]
MWCNT/GCE	Multi-walled carbon-nanotube-modified glassy carbon electrode	LSV	7	3–1200	0.9	n.r.	[48]
O-BDDP/SPE	Oxygen-terminated boron-doped diamond powder printed electrode	LSV	4	1–30	0.588	n.r.	[49]

n.r.: not reported. Tech.: DPV = differential pulse voltammetry; CA = chronoamperometry; AdSV-DPV = adsorptive stripping voltammetry with DPV readout; LSV = linear sweep voltammetry.

## Data Availability

The Python scripts used for electrochemical data processing, baseline correction, peak extraction, calibration fitting, and plotting are publicly available through Zenodo at https://doi.org/10.5281/zenodo.18668758. The experimental data supporting the findings of this study are available from the corresponding authors upon reasonable request.

## Supplementary Materials

The following supporting information can be downloaded at: https://www.mdpi.com/article/10.3390/s26217086/s1, Figure S1: Spectral irradiance of the UV–visible lamp measured at the electrode position (6 cm from the lamp) under the same geometrical conditions used for electrode regeneration. The spectrum is characterized by a dominant near-UV emission band centered at approximately 365 nm, together with discrete emission peaks in the visible region, confirming that the electrode was exposed to combined UV–visible irradiation during the regeneration treatment; Figure S2: Electrochemical impedance spectroscopy (EIS) characterization and equivalent circuit fitting for the investigated screen-printed carbon electrodes. For each panel, the top inset reports the best-fit equivalent circuit, while the bottom plot shows the corresponding Nyquist diagram (experimental points and fitted curve). (a) Bare SPE_C, (b) SPE_C_CNT, (c) SPE_C_HTA, (d) SPE_C_CNT-HTA, and (e) SPE_C_CNT-HTB. The fitted parameters (including $R_{s}$ and $R_{\mathrm{ct}}$) were used to compare the interfacial charge-transfer properties and to support the selection of the optimal sensing interface; Figure S3: Morphological characterization of the CNT/H_2_O sample via Scanning Electron Microscopy. (a) Low-magnification (86×) overview illustrating the macroscopic concentric morphology of the sample. The dashed boxes indicate the specific regions selected for high-resolution analysis. (b) High-magnification micrograph (50k×) of the outer ring area, showing a structural transition toward bright granular agglomerates and particulate features, morphologically distinct from the inner core. (c) High-magnification micrograph (50k×) of the central core region, revealing a dense, intertwined network of carbon nanotubes (CNTs) with a characteristic weave-like morphology; Figure S4: Electrochemical impedance spectroscopy (EIS) characterization of the CNT–halloysite hybrid interfaces fabricated on carbon screen-printed electrodes. The panel reports Nyquist plots (symbols) and the corresponding fits (solid lines) for SPE_C_CNT-HTA-X3, SPE_C_CNT-HTB-X3, SPE_C_CNT-HTA-X5, and SPE_C_CNT-HTB-X5. For each electrode, the best-fitting equivalent circuit is shown above the experimental data, consisting of the solution resistance ($R_{s}$) in series with a charge-transfer element ($R_{\mathrm{ct}}/R_{p}$) in parallel with a constant phase element (CPE) to account for non-ideal interfacial capacitance, and a Warburg element (W) to capture diffusion-controlled contributions at low frequencies. The extracted parameters highlight the effect of halloysite type and multilayer loading (X3 vs. X5) on interfacial charge-transfer and mass-transport features; Figure S5: Differential pulse voltammograms (DPV) recorded in PBS containing ciprofloxacin 34 μM under dark conditions for the investigated electrodes: bare carbon screen-printed electrode (SPE_C), CNT-modified electrode (SPE_CNT), halloysite-only electrode (SPE_HTA), and hybrid CNT/halloysite interfaces (SPE_CNT-HTA and SPE_CNT-HTB). Each panel reports the differential current response as a function of the applied potential, enabling a direct comparison of peak position and signal intensity across the different surface chemistries; Figure S6: UV–Vis absorption spectra of 34 μM ciprofloxacin (CIP) recorded after stabilization in the dark and after 10 min of UVA irradiation. Measurements were performed in the absence of halloysite nanotubes (HT), (a) and in the presence of HT (b). In the control solution containing only CIP, UVA exposure produced a negligible variation of the absorption band centered at ca. 276 nm, with a peak change of about 0.5%. Conversely, in the presence of HT, the same UVA treatment induced a more pronounced decrease of the absorption band at ca. 276 nm, corresponding to an approximately 6.6% relative reduction in the main absorption peak; Figure S7: SEM micrographs of the final SPE_C_CNT-HTA-X5 sensor under different conditions: (A) unused sensor, (B) used sensor after UVA-assisted cleaning, and (C) used sensor without UVA cleaning. All images were acquired at the same magnification (50.00k×); scale bar: 1 μm. The figure provides a qualitative morphological comparison of the electrode surface before use and after sensing/regeneration treatments; Figure S8: Differential pulse voltammogram of 8.5 μM tinidazole (TDZ) in PBS, recorded using the SPE_C_CNT-HTA-X5 electrode in the cathodic potential range (−1.0 to 0.0 V). The characteristic TDZ reduction feature is observed near −0.65 V versus the integrated Ag pseudo-reference, while the more prominent peak around −0.37 V is attributed to the reduction of oxygen. The TDZ reduction potential is well separated from the CIP oxidation peak (approximately +0.74–0.81 V), confirming the absence of direct overlap between their characteristic electrochemical signals; Figure S9: Uncorrected DPV responses (as acquired) recorded in CIP-free PBS after water rinsing alone (orange solid curve) and after a subsequent 10 min UVA treatment (blue dashed curve). Following UVA irradiation, the residual CIP-related oxidation feature is no longer resolved, suggesting a reduction of the residual analyte-related response. However, a change in background is observed in the irradiated sample. These changes may be due to repeated measurements in PBS (salt accumulation) and UVA-induced changes at the electrode-electrolyte interface (different surface charge). Since regeneration was evaluated using a single electrode, these results provide preliminary evidence of cleaning effectiveness, while reproducibility across different electrodes remains to be verified.

## Abbreviations

CIP	Ciprofloxacin
CNT	Carbon nanotube
HT	Halloysite nanotube
SPE_C	Carbon screen-printed electrode
DPV	Differential pulse voltammetry
EIS	Electrochemical impedance spectroscopy
SEM	Scanning electron microscopy
EDX	Energy-dispersive X-ray spectroscopy
PBS	Phosphate-buffered saline
LOD	Limit of detection
LOQ	Limit of quantification
UVA	Ultraviolet A
