# Supplementary material for: Eco-Friendly and Reusable CNT/Halloysite-Based Sensor for Electrochemical Ciprofloxacin Detection
Source: Sensors (Basel). 2026 Nov 9;26(21):7086. doi: 10.3390/s26217086 (PMC13766466; doi:10.3390/s26217086)
Supplement: Supplementary file 1 [file sensors-26-07086-s001.zip › sensors-4618495-supplementary.pdf]

Eco-friendly and reusable CNT/halloysite-Based Sensor for Electrochemical Ciprofloxacin Detection

Electronic Supplementary Information

Carlo Spampinato[a,*], Ivana Maria Caruso[a,b], Viviana Scuderi[a,*], Simona Filice[a], Andrzej Krasinski[c], Silvia Scalese[a]

a Consiglio Nazionale delle Ricerche - Istituto di Microelettronica e Microsistemi (CNR-IMM), VIII Strada, zona industriale, Catania, 95121, Italy,

b Università degli Studi di Catania, Dipartimento di Fisica ed Astronomia, Via Santa Sofia, n.64, Catania, 95125, Italy,

c Faculty of Chemical and Process Engineering, Warsaw University of Technology, 00-645, Warsaw, Poland.

*Corresponding author: carlo.spampinato@cnr.it; viviana.scuderi@cnr.it

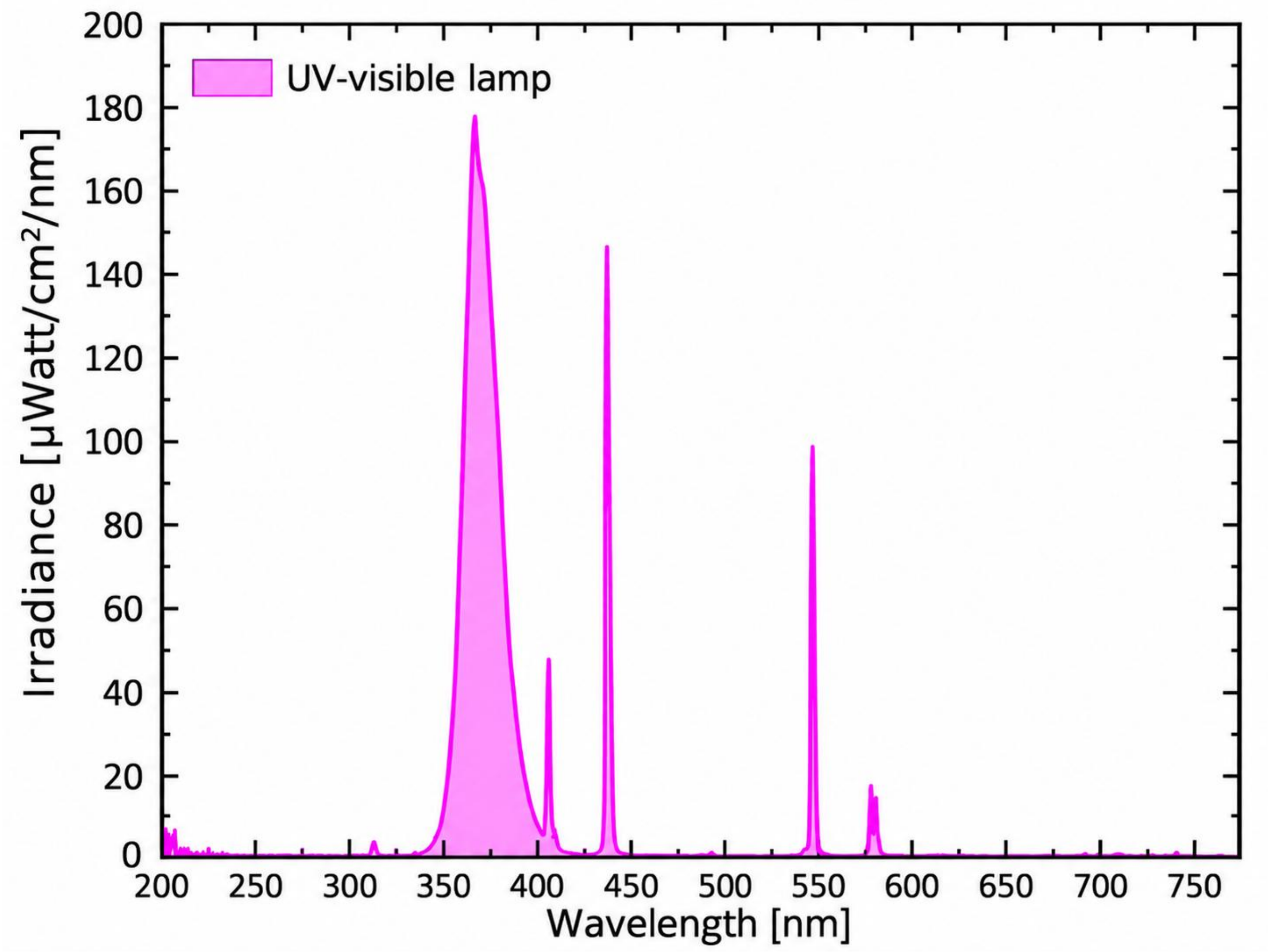

Figure S1: Spectral irradiance of the UV–visible lamp measured at the electrode position (6 cm from the lamp) under the same geometrical conditions used for electrode regeneration. The spectrum is characterized by a dominant near-UV emission band centered at approximately 365 nm, together with discrete emission peaks in the visible region, confirming that the electrode was exposed to combined UV–visible irradiation during the regeneration treatment.

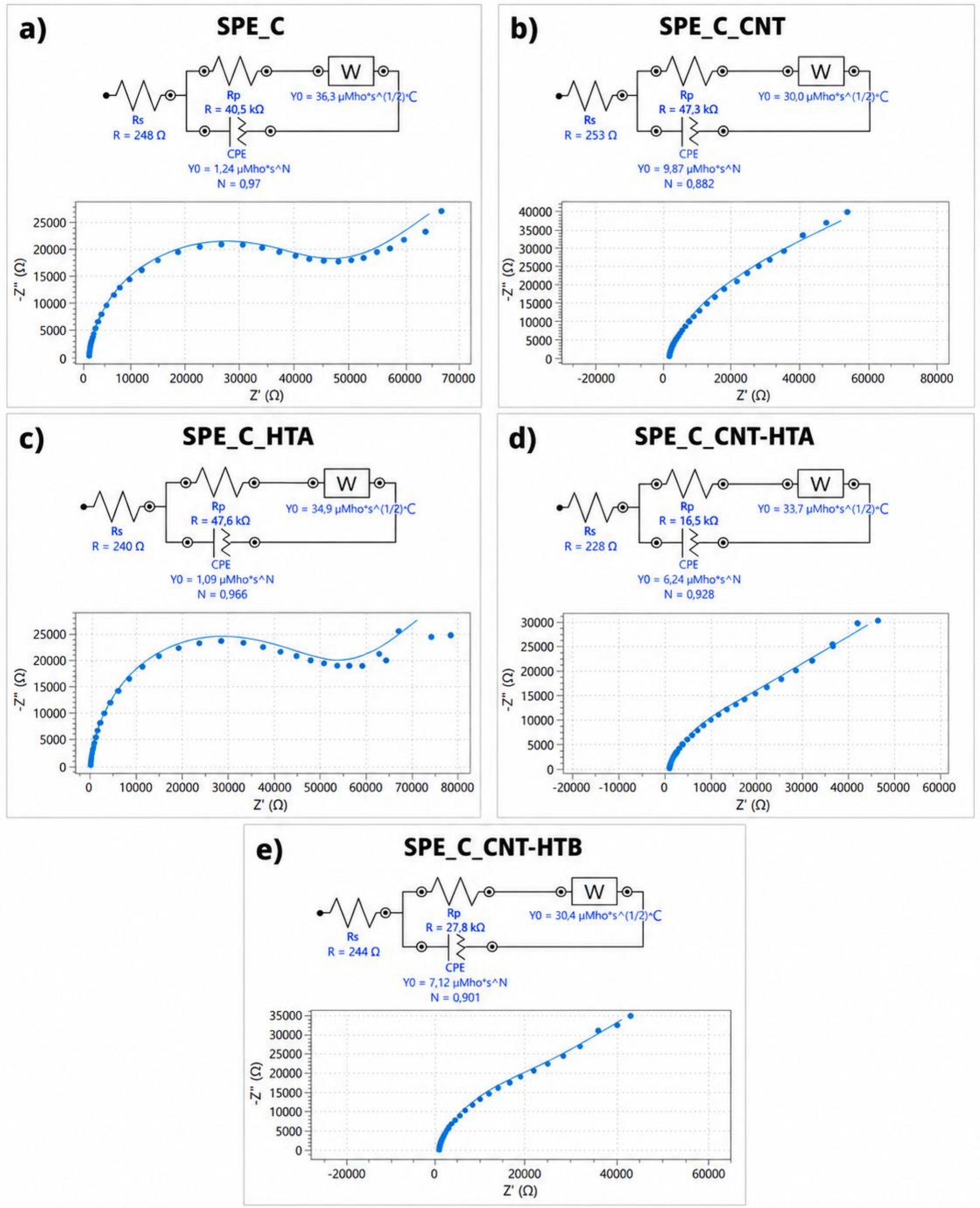

Figure S2: Electrochemical impedance spectroscopy (EIS) characterization and equivalent circuit fitting for the investigated screen-printed carbon electrodes. For each panel, the top inset reports the best-fit equivalent circuit, while the bottom plot shows the corresponding Nyquist diagram (experimental points and fitted curve). (a) Bare SPE_C, (b) SPE_C_CNT, (c) SPE_C_HTA, (d) SPE_C_CNT-HTA, and (e) SPE_C_CNT-HTB. The fitted parameters (including Rs and Rct) were used to compare the interfacial charge-transfer properties and to support the selection of the optimal sensing interface.

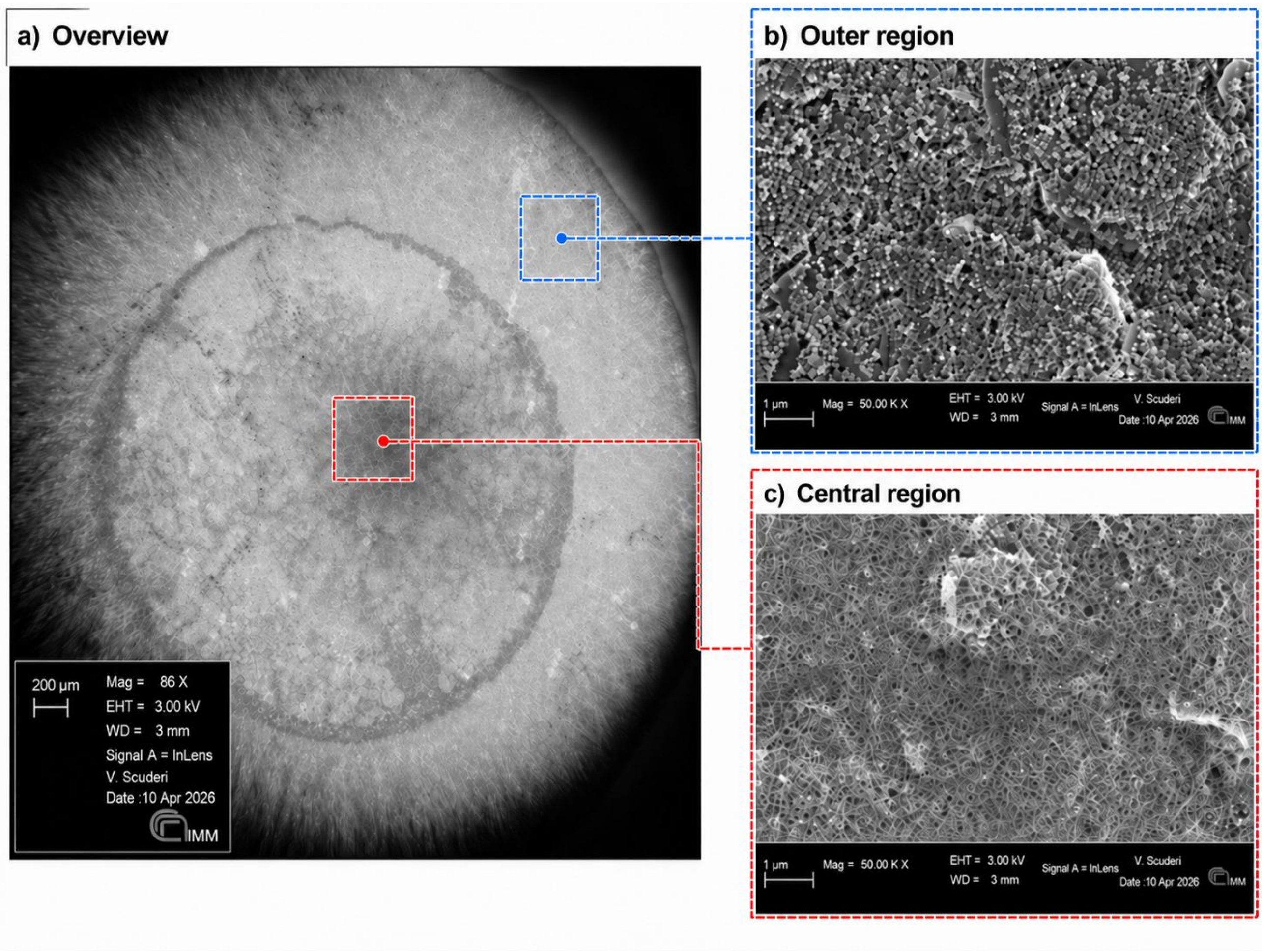

Figure S3: Morphological characterization of the CNT/H_2O sample via Scanning Electron Microscopy. (a) Low-magnification (86 X) overview illustrating the macroscopic concentric morphology of the sample. The dashed boxes indicate the specific regions selected for high-resolution analysis. (b) High-magnification micrograph (50 kX) of the outer ring area, showing a structural transition toward bright granular agglomerates and particulate features, morphologically distinct from the inner core. (c) High-magnification micrograph (50 kX) of the central core region, revealing a dense, intertwined network of carbon nanotubes (CNTs) with a characteristic weave-like morphology.

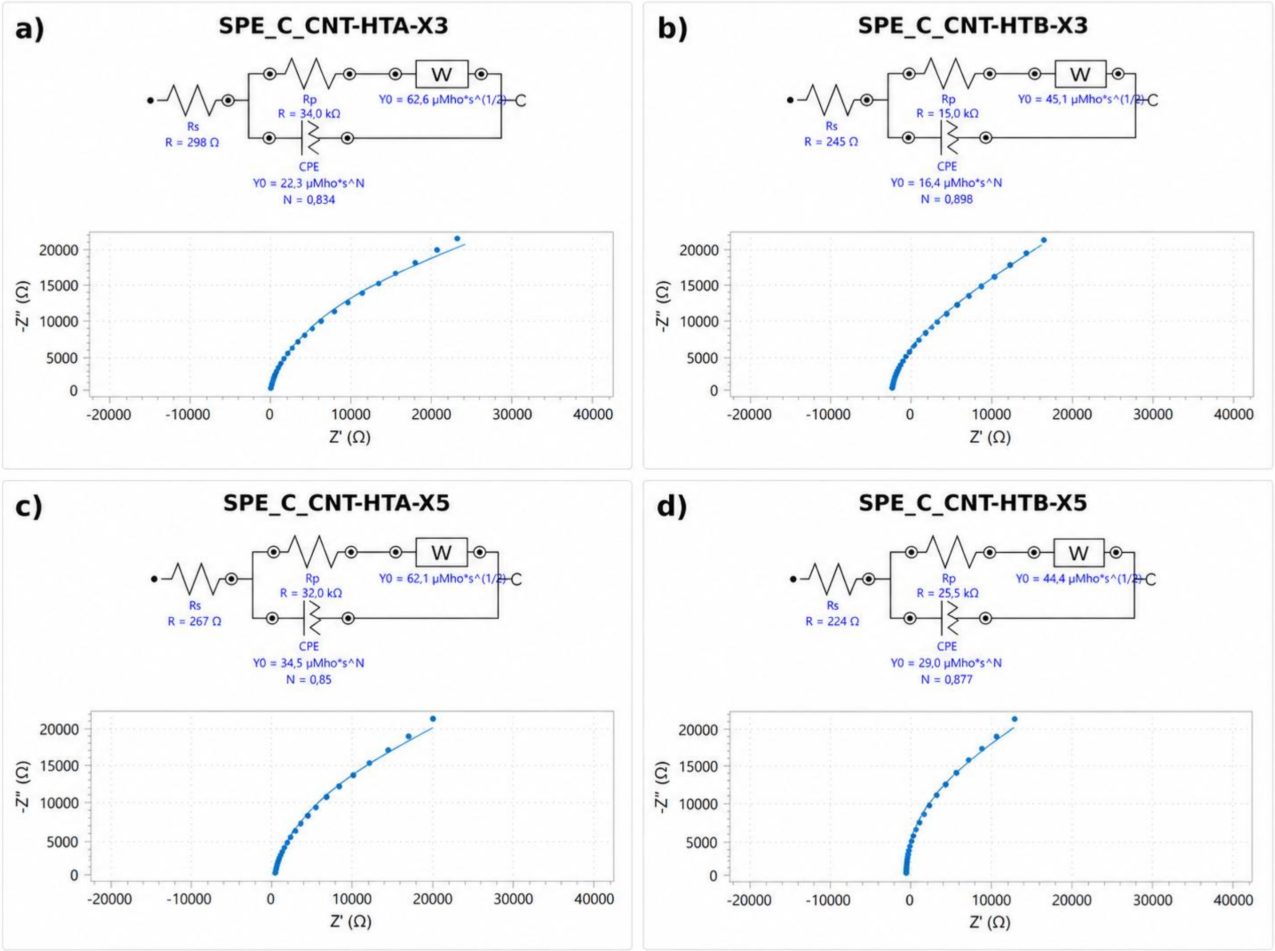

Figure S4: Electrochemical impedance spectroscopy (EIS) characterization of the CNT-halloysite hybrid interfaces fabricated on carbon screen-printed electrodes. The panel reports Nyquist plots (symbols) and the corresponding fits (solid lines) for SPE_C_CNT-HTA-X3, SPE_C_CNT-HTB-X3, SPE_C_CNT-HTA-X5, and SPE_C_CNT-HTB-X5. For each electrode, the best-fitting equivalent circuit is shown above the experimental data, consisting of the solution resistance (Rs) in series with a charge-transfer element (Rct/Rp) in parallel with a constant phase element (CPE) to account for non-ideal interfacial capacitance, and a Warburg element (W) to capture diffusion-controlled contributions at low frequencies. The extracted parameters highlight the effect of halloysite type and multilayer loading (X3 vs X5) on interfacial charge-transfer and mass-transport features.

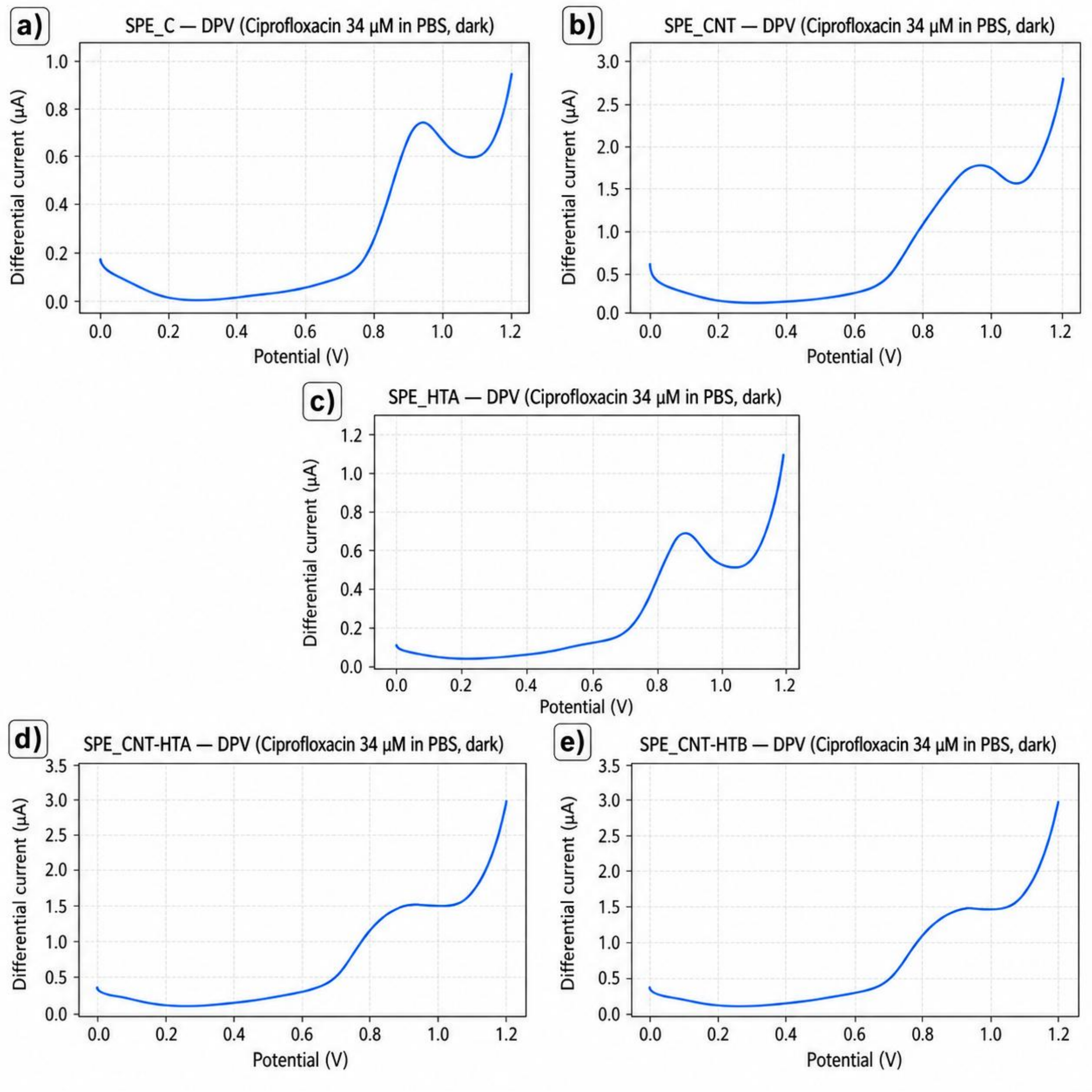

Figure S5: Differential pulse voltammograms (DPV) recorded in PBS containing ciprofloxacin 34µM under dark conditions for the investigated electrodes: bare carbon screen-printed electrode (SPE_C), CNT-modified electrode (SPE_CNT), halloysite-only electrode (SPE_HTA), and hybrid CNT/halloysite interfaces (SPE_CNT-HTA and SPE_CNT-HTB). Each panel reports the differential current response as a function of the applied potential, enabling a direct comparison of peak position and signal intensity across the different surface chemistries.

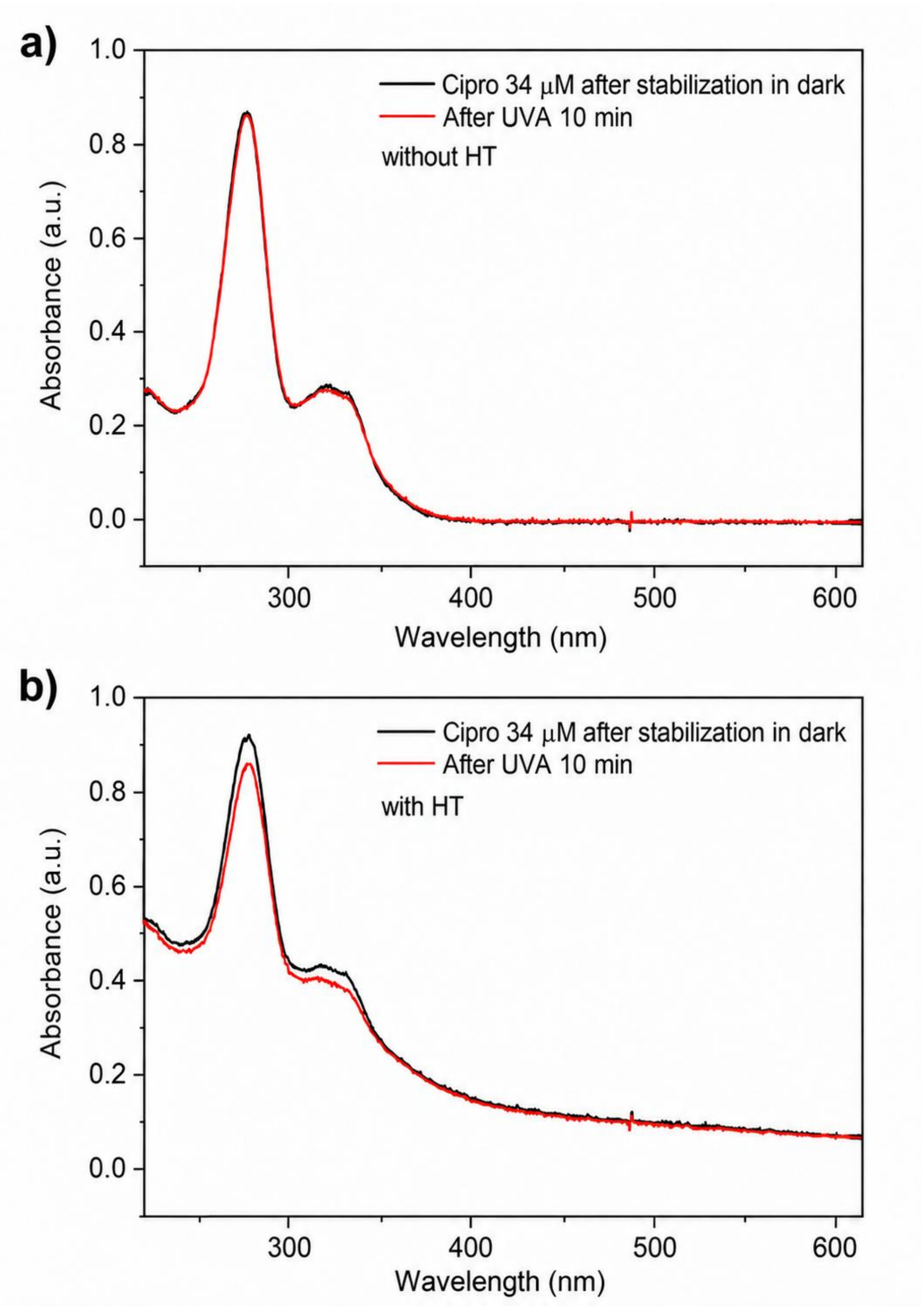

Figure S6: UV–Vis absorption spectra of 34 μM ciprofloxacin (CIP) recorded after stabilization in the dark and after 10 min of UVA irradiation. Measurements were performed in the absence of halloysite nanotubes (HT), (a) and in the presence of HT (b). In the control solution containing only CIP, UVA exposure produced a negligible variation of the absorption band centered at ca. 276 nm, with a peak change of about 0.5%. Conversely, in the presence of HT, the same UVA treatment induced a more pronounced decrease of the absorption band at ca. 276 nm, corresponding to an approximately 6.6% relative reduction in the main absorption peak.

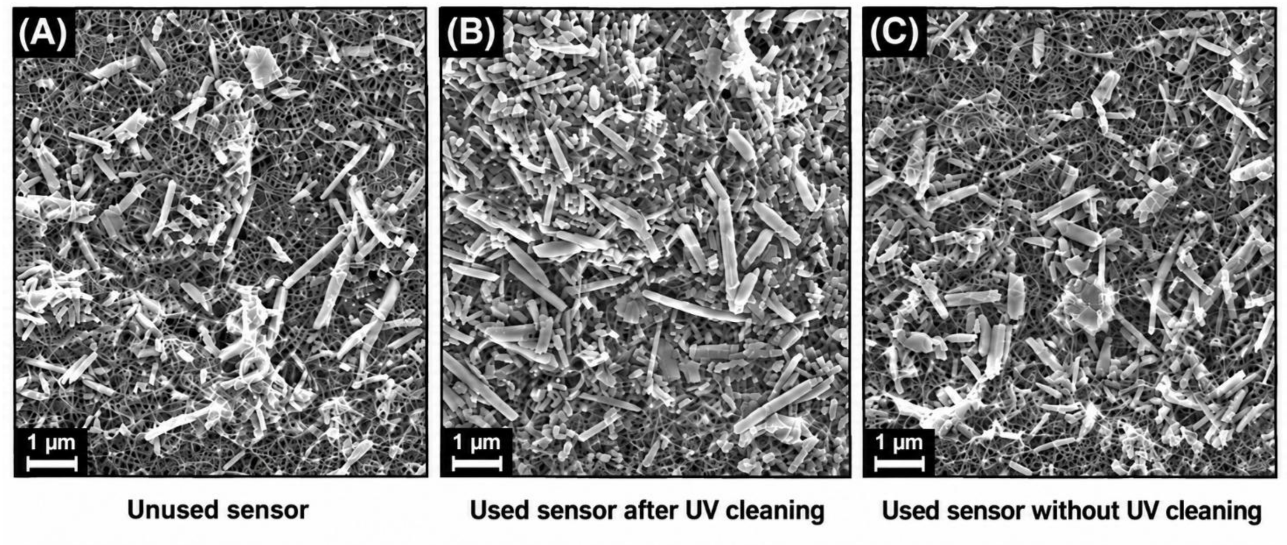

Figure S7. SEM micrographs of the final SPE_C_CNT-HTA-X5 sensor under different conditions: (A) unused sensor, (B) used sensor after UVA-assisted cleaning, and (C) used sensor without UVA cleaning. All images were acquired at the same magnification (50.00 kX); scale bar: 1 µm. The figure provides a qualitative morphological comparison of the electrode surface before use and after sensing/regeneration treatments.

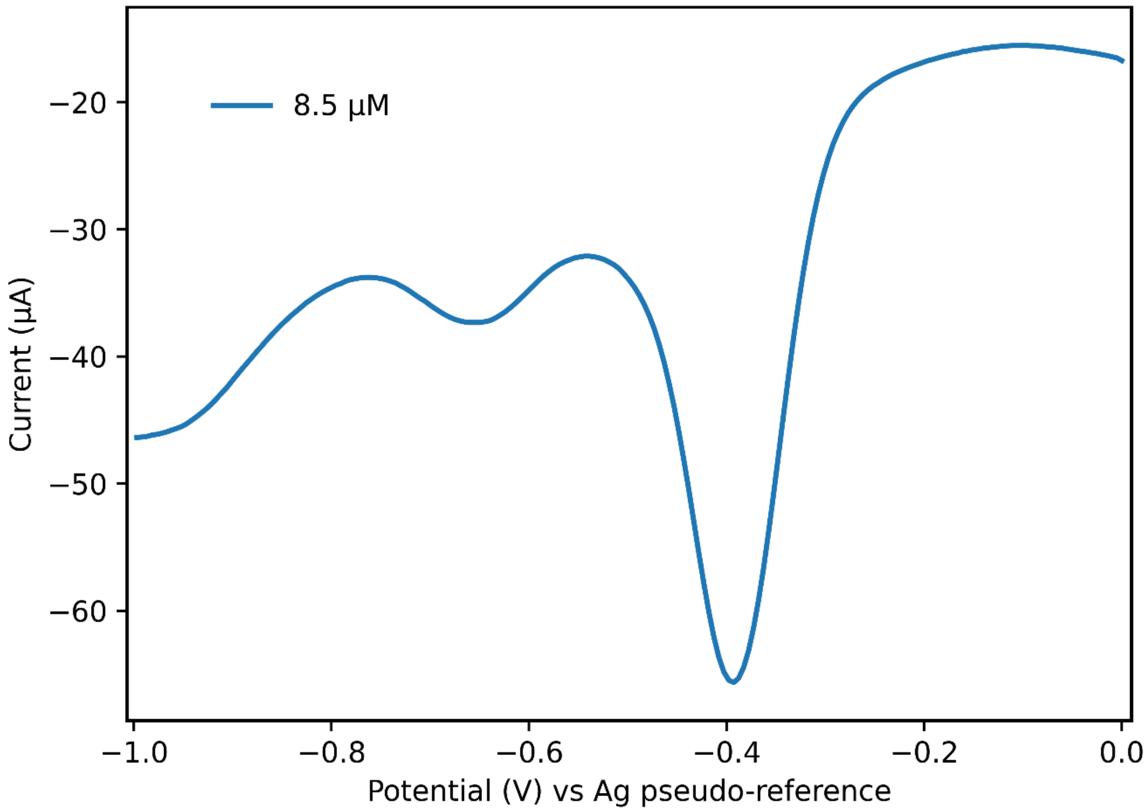

Figure S8. Differential pulse voltammogram of 8.5 μM tinidazole (TDZ) in PBS, recorded using the SPE_C_CNT-HTA-X5 electrode in the cathodic potential range (−1.0 to 0.0 V). The characteristic TDZ reduction feature is observed near −0.65 V versus the integrated Ag pseudo-reference, while the more prominent peak around −0.37 V is attributed to the reduction of oxygen. The TDZ reduction potential is well separated from the CIP oxidation peak (approximately +0.74–0.81 V), confirming the absence of direct overlap between their characteristic electrochemical signals.

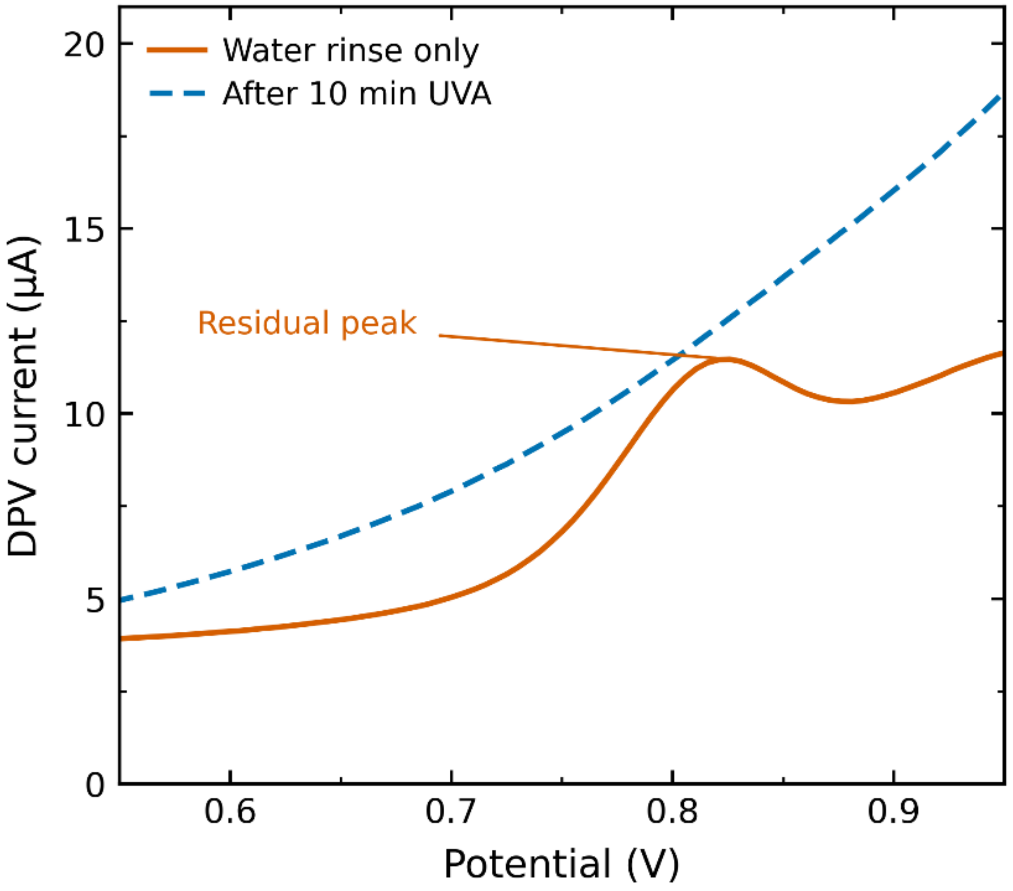

Figure S9. Uncorrected DPV responses (as acquired) recorded in CIP-free PBS after water rinsing alone (orange solid curve) and after a subsequent 10 min UVA treatment (blue dashed curve). Following UVA irradiation, the residual CIP-related oxidation feature is no longer resolved, suggesting a reduction of the residual analyte-related response. However, a change in background is observed in the irradiated sample. These changes may be due to repeated measurements in PBS (salt accumulation) and UVA-induced changes at the electrode-electrolyte interface (different surface charge). Since regeneration was evaluated using a single electrode, these results provide preliminary evidence of cleaning effectiveness, while reproducibility across different electrodes remains to be verified.
